# Canadians Think that Nearly All of Us Will Be Allowed Back to Work around August

**DOI:** 10.1017/S0008423920000487

**Published:** 2020-05-15

**Authors:** Ryan C. Briggs

**Affiliations:** Guelph Institute of Development Studies and Department of Political Science, University of Guelph, 50 Stone Road East, Guelph, ON N1G 2W1, Canada

## Abstract

Business closures and work-from-home orders have been a central part of Canada's plan to slow the spread of COVID-19. The success of these measures hinges on public support, which cannot be taken for granted as the orders induce considerable economic pain. As governments consider when to re-open the economy, one relevant variable is when the public expects the economy to re-open. At minimum, if public perceptions differ from government plans then additional government messaging is required to better align expectations.

Business closures and work-from-home orders have been a central part of Canada's plan to slow the spread of COVID-19. The success of these measures hinges on public support, which cannot be taken for granted as the orders induce considerable economic pain. As governments consider when to re-open the economy, one relevant variable is when the public expects the economy to re-open. At minimum, if public perceptions differ from government plans then additional government messaging is required to better align expectations.

We currently have little information on when the Canadian public expects the economy to re-open. I provide an answer to this question using a recent national survey and multilevel regression and poststratification (MRP). This method allows me to correct for biases in the survey sample and to estimate public opinion at much smaller levels of aggregation and with lower variance than is possible with normal methods. I show that most groups of Canadians expect us to be back to work by around early August, and very few people expect us to be back to work before July.

## Method

I make use of survey data originally collected in Sevi et al. (2020), which tested the effects of various graphical presentations of COVID-19 spread on public support for closures of the economy. This article uses the same data but describes public opinion instead of testing a causal effect.^1^

The key outcome variable is the answer to the question of when the respondent expects the government to allow nearly everyone back to work. The survey was conducted April 3–5, 2020, and the answers are integers ranging in increasing months from 1 to 11, where 1 = April and the numbers increase evenly to 9 = December, with 10 being “sometime in 2021” and 11 being “never.” The vast majority of answers are in the 1–9 range, and I model the outcome as a count using MRP, a method that draws on work in Gelman and Little (1997) and Park et al. (2004). In the graphical presentations, I treat each integer as representing the first of the associated month.

MRP has been used to estimate subnational public opinion on a wide range of issues from climate change in Canada (Mildenberger et al., 2016) to same sex marriage in the United States (Warshaw and Rodden, 2012). MRP generally performs best when it makes use of geographic covariates of public opinion (Hanretty et al., 2018; Warshaw and Rodden, 2012), and even under non-ideal circumstances it tends to outperform other methods of estimating subgroup opinion such as simple disaggregation. Even more critical studies of MRP “agree that its performance exceeds that of other methods” (Buttice and Highton, 2013: 464).

MRP is composed of two steps, a modeling step and a poststratification step. In the modeling step, I estimate the relationship between individual and geographic covariates and the outcome of interest using a multilevel model. In the poststratification step, I take predictions from this model for each demographic-geographic subgroup and re-weight them based on their share of the population in the unit of interest.^2^ Combining these two steps allows me to estimate public opinion at finer levels and with less variance than is practical with even large national surveys.

I use this approach to estimate when the Canadian public expects the economy to re-open, and I do this at the level of the country, province, and federal electoral districts, and for the demographic groupings of age, gender, and education. I first model each individual's response as a function of his or her demographic characteristics and location. For individual *i*, with indexes *j*, *k*, *l*, *m*, for age by gender categories, level of education, federal electoral district, and region, respectively:
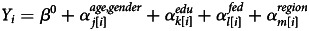
where the terms after the intercept are modelled as coming from a normal distribution with mean zero:
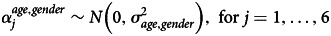

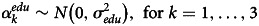


Federal electoral districts are modelled as inside regions with the following geographic covariates measured at the level of the riding: liberal vote share in the 2019 election, mean income per tax filer in 2017, Canadian childcare benefits per recipient, population, and population density. The variables are aimed at measuring political attitudes in an area, the area's economic status, and its rurality. More formally:
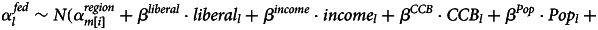

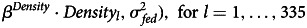


Finally, regions are provinces in all cases, except that Manitoba and Saskatchewan are combined and the Maritime provinces are combined with each other and with Newfoundland. In both cases, this was done to allow pooling of information across provincial boundaries in provinces that had small numbers of observations. I do not generate predictions for the territories. Regions are modelled with only one geographic covariate, which is the total number of COVID-19 cases reported in the province as of April 3, 2020 (Dong et al., 2020):
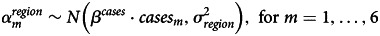


The model is fit using the stan_glmer function in the rstanarm package, which is simply a wrapper for Stan (Carpenter et al., 2017). I use the default, weakly informative priors and the Poisson family. Similar point estimates can be obtained using the glmer function in lme4.^3^

The next step is poststratification, which is when predictions for each demographic-geographic type are generated and then weighted by the percentage of that type in the aggregate unit of interest. There are two gender types, three education types, three age groupings, and 335 federal electoral districts for a total of 6,030 different subgroups. To generate a prediction of public opinion at the provincial level, for example, one draws a prediction for all 6,030 subgroups and then weights each one by its share of the population within each province. Formally, I draw from the posterior predictive distribution for each subgroup *c* a prediction of when the economy will re-open, *ϑc*. Each subgroup also has a population count, *Nc*. I then weight each cell's prediction by its share of the total population of the relevant group, *g*:
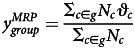


I repeat this process 10,000 times and report either mean predictions per group or the full distribution of predictions across all 10,000 draws.

## Results

**Figure 1.**
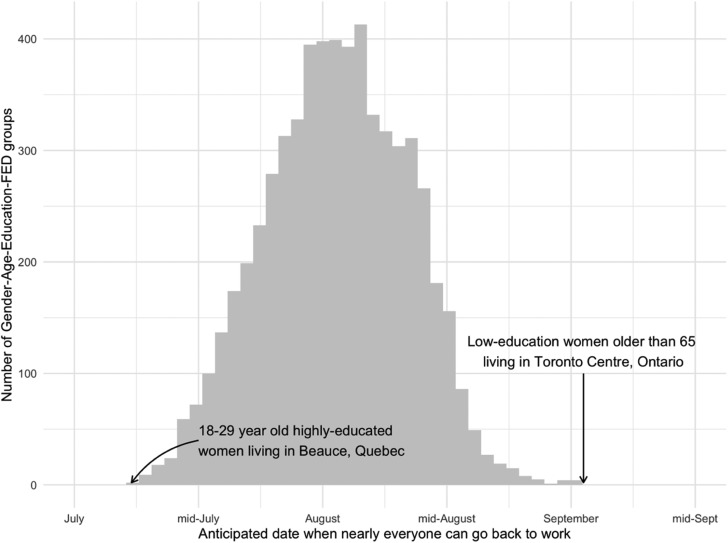
Distribution of the mean estimate for each of the 6,030 subgroups.

Figure 1 shows the distribution of the estimated date of re-opening across all gender × age × education × electoral district groupings.^4^ This shows the results of the multilevel model before poststratification. Most of the groups expect Canadians to be back to work by around August, and none of them expects the economy to re-open before July. The remaining analyses show poststratified results.

**Figure 2.**
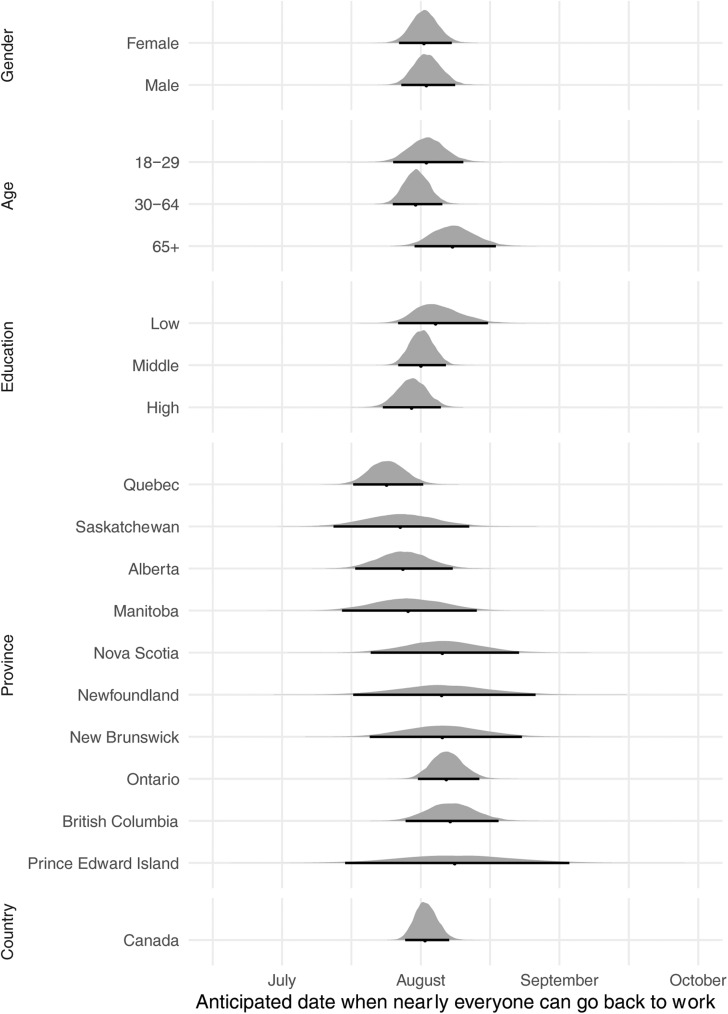
Poststratified estimates.

Figure 2 shows the results after poststratifying the predictions. Poststratification adjusts the predictions so that each subgroup's prediction's weight is proportionate to its population share. This means that the female group incorporates the fact that women tend to be older than men, for example. The figures show the distribution of estimates from 10,000 draws from the model as well as the central estimate and a 95 percent interval. Provinces with more observations, like Ontario, have more precise estimates than provinces like PEI. No group expects the economy to reopen before July or after September. The minimum bottom percentile across all of the distributions in Figure 2 belongs to Saskatchewan and is 4.3, where 4 represents July. The 98 percent prediction interval for Canada as-a-whole runs from 4.9 to 5.2, where 5 represents August.

**Figure 3.**
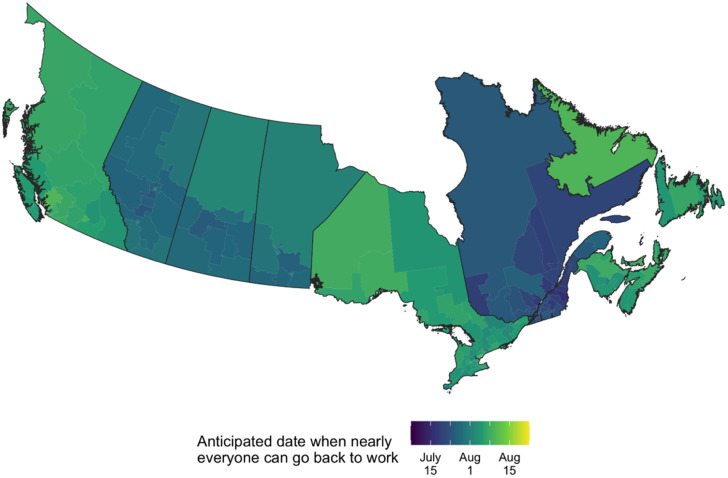
FED-level estimates.

**Figure 4.**
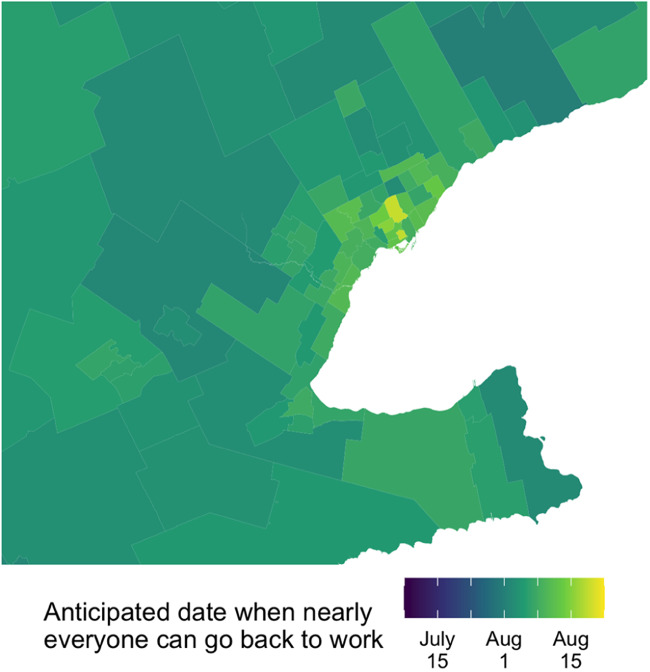
Ontario's Golden Horseshoe.

**Figure 5.**
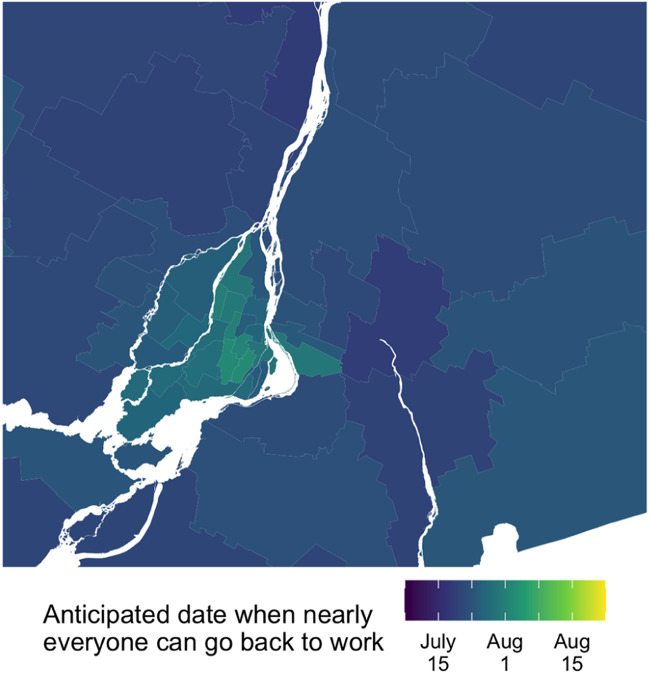
Montreal.

Figures 3–5 map the mean estimates for each federal electoral district. While the model has a lot of uncertainty at this level of disaggregation, the map suggests that people in cities expect to be back to work later than people living in more rural parts of Canada. This effect holds in a province that expects the economy to re-open later, like Ontario, and also in a province that expects the economy to re-open earlier, like Quebec.

The three largest caveats in this article are that it relies on only one sample of about 2,500 Canadians, that it assumes that people understood the question and used it to express an opinion rather than just guessing at the middle of the range of possible answers,^5^ and that it could have more powerful geographic predictors. Future work could address these issues, and it could also consider more flexible methods of regularization (Bisbee, 2019).

## Conclusion

It is important to understand how long Canadians expect mass workplace closures to last. This note analyzed a national survey with about 2,500 people using MRP and showed that most Canadians think that we will not be back to work before July. The vast majority of Canadians do however expect us to be back to work before the start of September. Differences across demographic groups and provinces are noticeable but relatively small. Quebecers and those with higher education expect an earlier re-opening, while older Canadians and residents of Ontario and British Columbia expect a later re-opening.

Finally, it is worth noting that Canada-wide support for workplace closure policies is almost uniformly high despite Canadians expecting the economy to stay closed until early August (Sevi et al., 2020).^6^ This is quite an impressive achievement, for both the government and the public.

## Notes

1 Figure 2 of Sevi et al. (2020) presented subgroup averages for expected duration of economy-wide shutdown for a number of groups, but these came from unweighted regressions using data that are quite far from the Canadian population on some measures. For example, only 5 per cent of the sample but over 40 per cent of Canada's population is in the “low education” category. Attention to these issues is generally superfluous when testing causal effects, but it is vital for description.

2 To do this, I use information on the number of people in Canada by age by gender by level of education within every Federal electoral district. This is available from StatsCan, Table 98-400-X2016243.

3 The Poisson model yields very similar estimates to a negative binomial model but converges more easily. See the Supplementary Information (available online) for more detail on the modeling.

4 Each subgroup prediction is an average across 10,000 draws from the posterior predictive distribution.

5 See footnote 5 in Sevi et al. (2020).

6 The rate of support is so uniformly high that it is not worth modeling. For example, 54 per cent of Canadians picked 10 (out of 10) for their support for the government's stay at home order, 70 per cent picked 9 or 10, and 85 per cent picked 8 or above. Less than 3 per cent picked below 5.

## Supplementary material

For supplementary material accompanying this paper visit http://dx.doi.org/10.1017/S0008423920000487. click here to view supplementary material
