# Supplementary material for: Canadians Think that Nearly All of Us Will Be Allowed Back to Work around August
Source: Can J Polit Sci. 2020 May 15:1–7. doi: 10.1017/S0008423920000487 (PMC7256219; doi:10.1017/S0008423920000487)
Supplement: Supplementary file 1 [file S0008423920000487sup001.pdf]

Supplementary Information for: Canadians think that nearly all of us will be allowed back to work around August

Ryan C. Briggs, rbriggs@uoguelph.ca

May 11, 2020

This notebook uses multilevel regression and poststratification to estimate when Canadians expect that nearly everyone will be able to go back to work again. It shows all of the analysis underlying the paper "Canadians think that nearly all of us will be allowed back to work around August." It also shows a number of related additional analyses.

Load libraries

```
#start timer
begin_time <- proc.time()

library(tidyverse)
```

```
## -- Attaching packages ------------------------------------------------------ tidyverse 1.3.0 --
## v ggplot2 3.3.0     v purrr   0.3.4
## v tibble  3.0.0     v dplyr   0.8.5
## v tidyr   1.0.2     v stringr 1.4.0
## v readr   1.3.1     v forcats 0.5.0
## Warning: package 'tibble' was built under R version 3.6.2
## Warning: package 'purrr' was built under R version 3.6.2
## -- Conflicts --------------------------------------------------------- tidyverse_conflicts() --
## x dplyr::filter() masks stats::filter()
## x dplyr::lag()    masks stats::lag()
```

```
library(lme4)
```

```
## Warning: package 'lme4' was built under R version 3.6.2
## Loading required package: Matrix
##
## Attaching package: 'Matrix'
## The following objects are masked from 'package:tidyr':
##
##     expand, pack, unpack
```

```
library(sf)
```

```
## Warning: package 'sf' was built under R version 3.6.2
## Linking to GEOS 3.7.2, GDAL 2.4.2, PROJ 5.2.0
```

```
library(rstanarm)
```

```
## Loading required package: Rcpp
## rstanarm (Version 2.19.3, packaged: 2020-02-11 05:16:41 UTC)
## - Do not expect the default priors to remain the same in future rstanarm versions.
## Thus, R scripts should specify priors explicitly, even if they are just the defaults.
## - For execution on a local, multicore CPU with excess RAM we recommend calling
## options(mc.cores = parallel::detectCores())
## - bayesplot theme set to bayesplot::theme_default()
##    * Does _not_ affect other ggplot2 plots
##    * See ?bayesplot_theme_set for details on theme setting
```

```
library(tidybayes)
```

```
## Warning: package 'tidybayes' was built under R version 3.6.2
```

```
#set cores for parallel processing
options(mc.cores = parallel::detectCores())

#load data for analysis
dat <- readRDS("dat.rds")
#load dataset for poststratification
poststrat <- readRDS("poststrat.rds")
#load shape file of federal electoral districts
fed <- st_read("Data/lfed000b16a_e/lfed000b16a_e.shp", stringsAsFactors = F)
```

```
## Reading layer `lfed000b16a_e' from data source `/Users/ryan/Dropbox/School/Articles in progress/Covi
## Simple feature collection with 338 features and 6 fields
## geometry type:  MULTIPOLYGON
## dimension:      XY
## bbox:           xmin: 3689439 ymin: 659338.9 xmax: 9015737 ymax: 5242179
## CRS:            3347
```

Descriptive analysis

The following block performs a simple descriptive analysis of the data. I first examine the distribution of the key outcome. Then I compare the sample to the Canadian population on three dimensions (age, sex, education). The sample is well balanced of the first two but badly balanced on education. Poststratification will, among other things, address this kind of imbalance.

```
#distribution of outcome by region of Canada
dat %>%
  ggplot(aes(x = as.factor(Duration), fill = Education)) +
  geom_histogram(stat = "count") +
  theme_minimal()
```

```
## Warning: Ignoring unknown parameters: binwidth, bins, pad
```

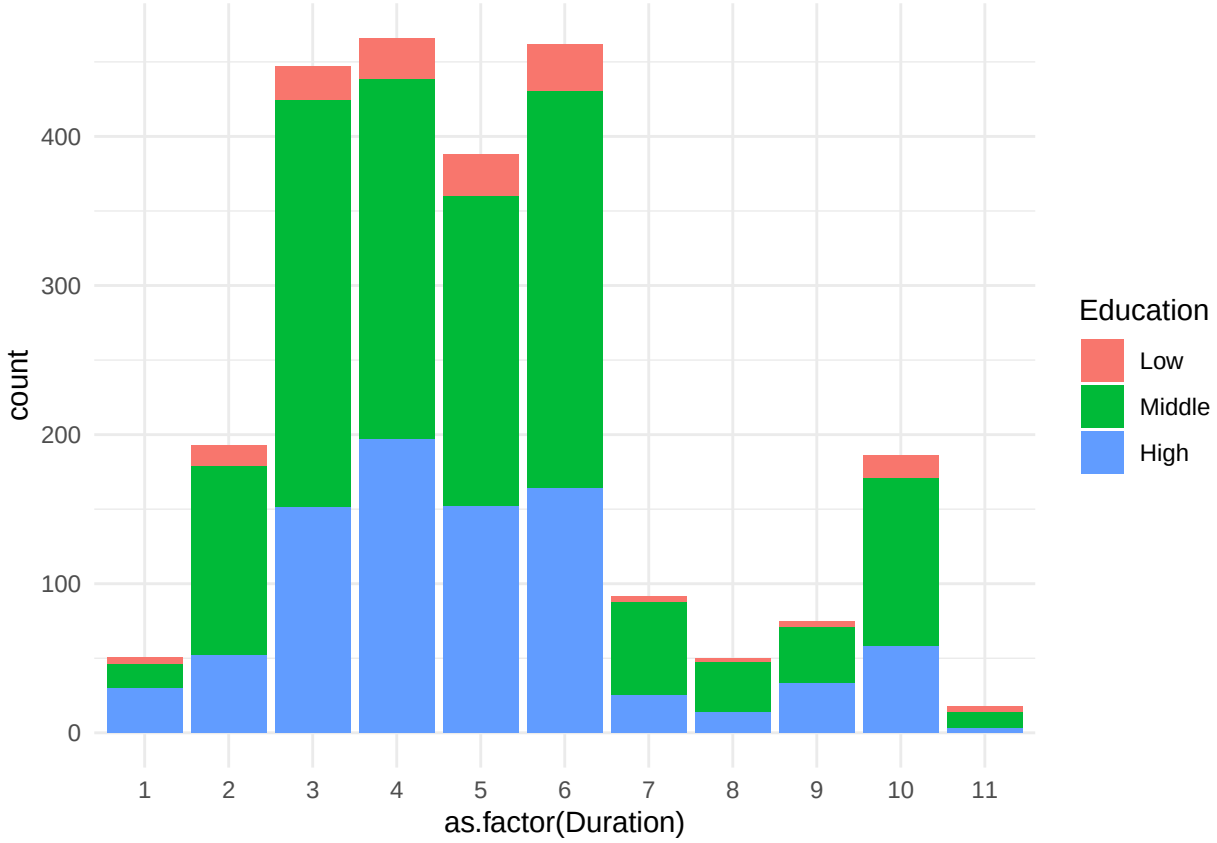


```
#fraction of people at different levels of support
round(table(dat$Support) / nrow(dat), 2)
```

```
##
##    0    1    2    3    4    5    6    7    8    9   10
## 0.00 0.00 0.00 0.01 0.01 0.03 0.03 0.08 0.14 0.16 0.54
```

```
#Checks of sample balance
#Relative to the population, the sample wildly underestimates people with low education
pop_edu <- poststrat %>%
  group_by(Education) %>%
  summarize(Total = sum(Total)) %>%
  ungroup() %>%
  mutate(Percent = Total / sum(Total),
         Data = "Population")

dat_edu <- dat %>%
  group_by(Education) %>%
  summarize(Total = n()) %>%
  ungroup() %>%
  mutate(Percent = Total / sum(Total),
         Data = "Sample")

rbind(pop_edu, dat_edu) %>%
  ggplot(aes(x = Education, y = Percent, fill = Data)) +
  geom_col(position = "dodge") +
  scale_fill_discrete(name = "") +
  theme_minimal() +
```

```
  theme(panel.grid.major.x = element_blank()) +
  ggsave("Education_balance.pdf", width = 6, height = 6)
```

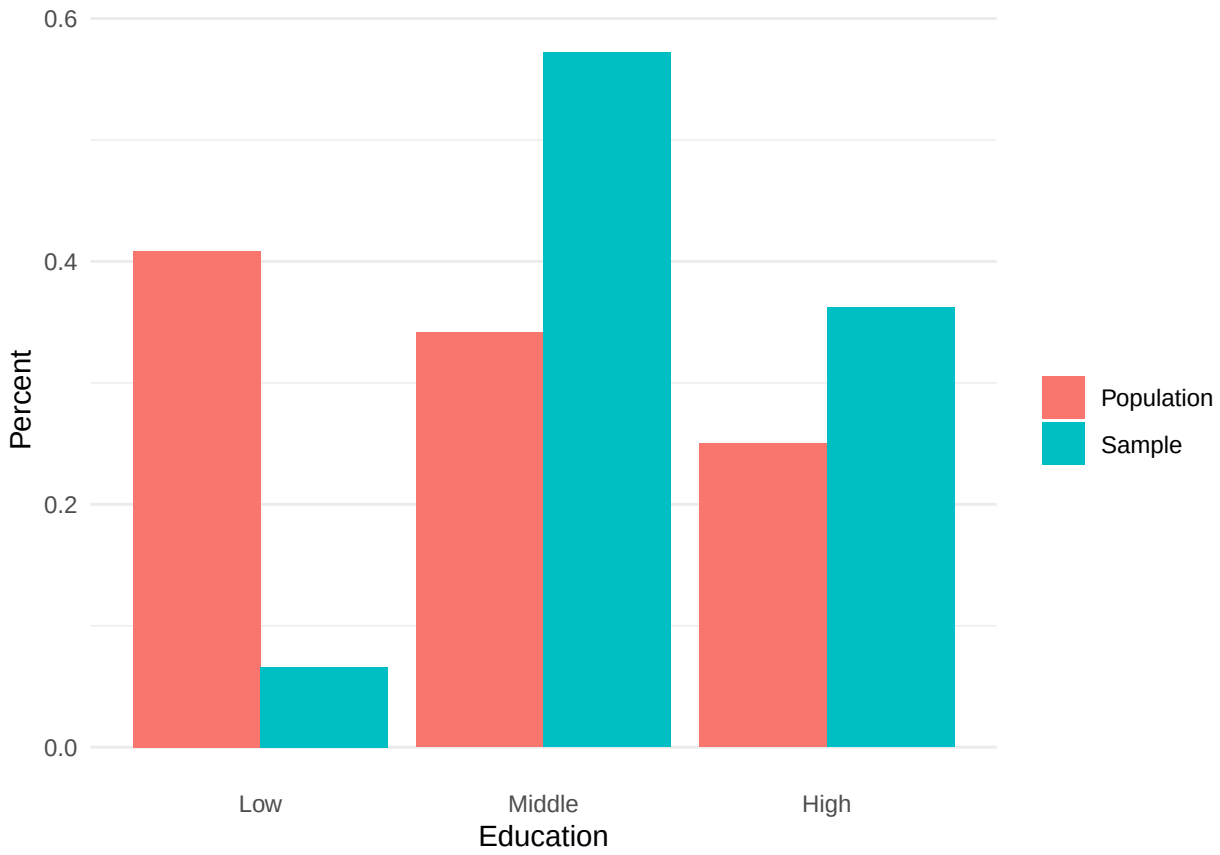


```
rm(pop_edu, dat_edu)

#Age balance is good, older men are a little over-represented
#younger men are a little under-represented
pop_age <- poststrat %>%
  group_by(Age, Gender) %>%
  summarize(Total = sum(Total)) %>%
  ungroup() %>%
  mutate(Percent = Total / sum(Total),
         Data = "Population")

dat_age <- dat %>%
  group_by(Age, Gender) %>%
  summarize(Total = n()) %>%
  ungroup() %>%
  mutate(Percent = Total / sum(Total),
         Data = "Sample")

rbind(pop_age, dat_age) %>%
  ggplot(aes(x = Age, y = Percent, fill = Data)) +
  geom_col(position = "dodge") +
  facet_wrap(~ Gender) +
  scale_fill_discrete(name = "") +
  theme_minimal() +
```

```
  theme(panel.grid.major.x = element_blank()) +
  ggsave("Age_balance.pdf", width = 8, height = 6)
```

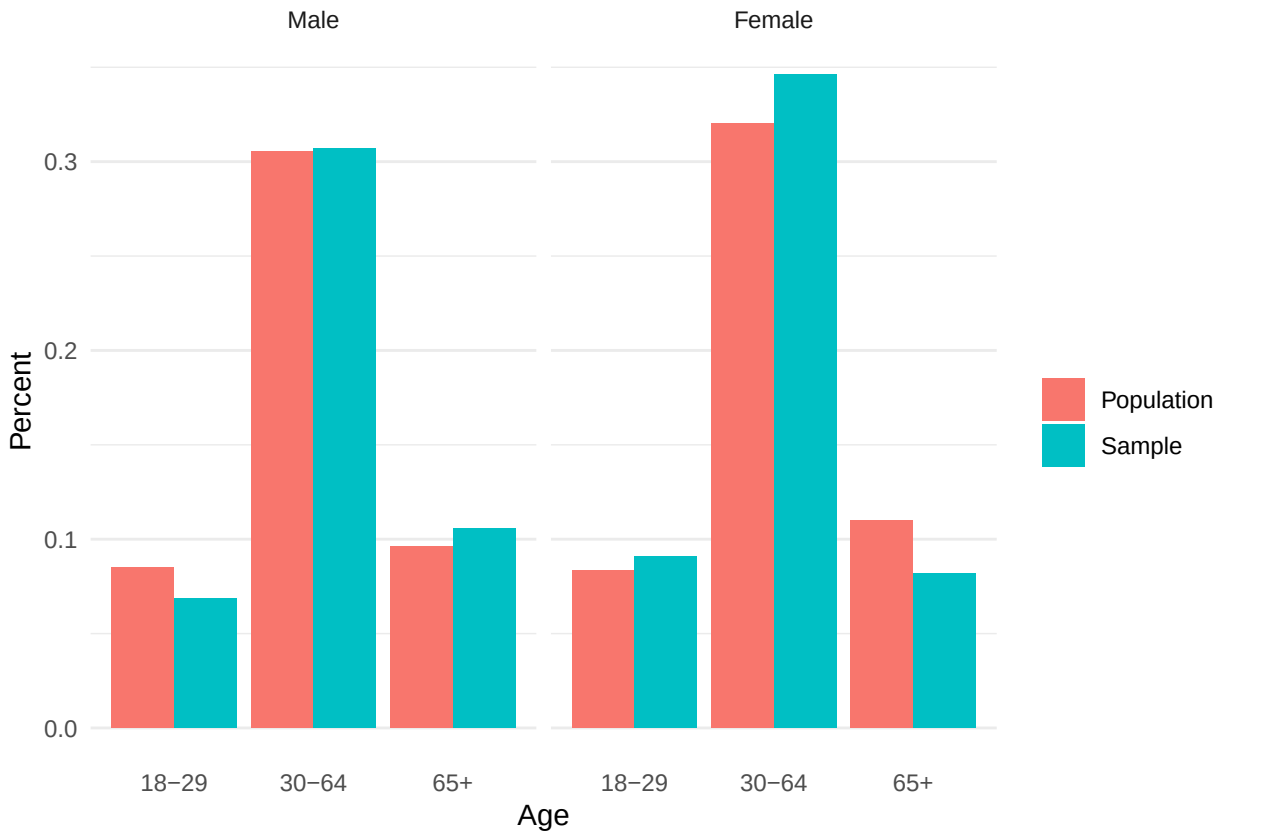


```
rm(pop_age, dat_age)
```

Multilevel regression

This next block does the multilevel regression. I fit both a GLM and a bayesian model. In the analysis, I make use of only the bayesian model but it is useful to see that two produce similar point estimates.

```
set.seed(2684319)

#scale and center variables used in the model and those used in the poststratification
vars <- c("Liberal_voteshare", "Inc_per_filer", "CCB_dollars_per_recip",
          "Population", "Pop_density", "Land_area", "Total_CCB_recipients",
          "Present_cases", "Week_before_cases")
dat <- mutate_at(dat, vars, scale)
poststrat <- mutate_at(poststrat, vars, scale)

#duration model via maximum likelihood
duration_mod <- glmer(formula = Duration ~ (1|Age_Gender) + (1|Education) +
                        (1|Region/FEDUID) + Liberal_voteshare + Inc_per_filer +
                        CCB_dollars_per_recip + Population + Pop_density +
                        Present_cases,
                      data = dat, family = poisson(link = "log"))

summary(duration_mod)
```

```
## Generalized linear mixed model fit by maximum likelihood (Laplace
##   Approximation) [glmerMod]
##  Family: poisson  ( log )
## Formula: Duration ~ (1 | Age_Gender) + (1 | Education) + (1 | Region/FEDUID) +
##     Liberal_voteshare + Inc_per_filer + CCB_dollars_per_recip +
##     Population + Pop_density + Present_cases
##    Data: dat
##
##      AIC      BIC   logLik deviance df.resid
##  10655.6  10719.4  -5316.8  10633.6     2417
##
## Scaled residuals:
##     Min      1Q  Median      3Q     Max
## -1.8630 -0.7904 -0.1164  0.4653  2.9819
##
## Random effects:
##  Groups        Name        Variance  Std.Dev.
##  FEDUID:Region (Intercept) 0.0003195 0.01787
##  Region        (Intercept) 0.0007843 0.02800
##  Age_Gender    (Intercept) 0.0012624 0.03553
##  Education     (Intercept) 0.0002538 0.01593
## Number of obs: 2428, groups:
## FEDUID:Region, 329; Region, 6; Age_Gender, 6; Education, 3
##
## Fixed effects:
##                        Estimate Std. Error z value Pr(>|z|)
## (Intercept)            1.610022   0.024987  64.435   <2e-16 ***
## Liberal_voteshare      0.019467   0.013171   1.478    0.139
## Inc_per_filer          0.009063   0.012377   0.732    0.464
## CCB_dollars_per_recip  0.007413   0.011977   0.619    0.536
## Population            -0.003411   0.011458  -0.298    0.766
## Pop_density            0.006989   0.010657   0.656    0.512
## Present_cases         -0.019619   0.016178  -1.213    0.225
## ---
## Signif. codes:  0 '***' 0.001 '**' 0.01 '*' 0.05 '.' 0.1 ' ' 1
##
## Correlation of Fixed Effects:
##             (Intr) Lbrl_v Inc_p_ CCB___ Popltn Pp_dns
## Librl_vtshr  0.093
## Inc_per_flr  0.001 -0.071
## CCB_dllrs__ -0.001 -0.076  0.590
## Population   0.076  0.177 -0.142  0.048
## Pop_density -0.006 -0.384 -0.211 -0.143 -0.061
## Present_css  0.101 -0.262  0.243  0.171 -0.259 -0.046
```

```r
#------------------
#Bayesian analysis, default priors normal (0, 2.5)
#A negative binomial yields very similar results, but struggles with convergence
#To run a NB, swap "poisson" for "neg_binomial_2(link = "log")"
duration_mod_stan <-
  stan_glmer(formula = Duration ~ (1|Age_Gender) + (1|Education) + (1|Region/FEDUID) +
               Liberal_voteshare + Inc_per_filer + CCB_dollars_per_recip + Population +
               Pop_density + Present_cases,
             #chains = 1, iter = 200, #for testing
```

```
             chains = 6, iter = 4000, #for real
             data = dat, family = poisson,
             seed = 2684319, adapt_delta = .9999)
```

```
## Warning: There were 9 divergent transitions after warmup. Increasing adapt_delta above 0.9999 may hel
## http://mc-stan.org/misc/warnings.html#divergent-transitions-after-warmup
```

```
## Warning: Examine the pairs() plot to diagnose sampling problems
```

```
duration_mod_stan
```

```
## stan_glmer
##  family:       poisson [log]
##  formula:      Duration ~ (1 | Age_Gender) + (1 | Education) + (1 | Region/FEDUID) +
##     Liberal_voteshare + Inc_per_filer + CCB_dollars_per_recip +
##     Population + Pop_density + Present_cases
##  observations: 2428
## ------
##                       Median MAD_SD
## (Intercept)           1.6    0.1
## Liberal_voteshare     0.0    0.0
## Inc_per_filer         0.0    0.0
## CCB_dollars_per_recip 0.0    0.0
## Population            0.0    0.0
## Pop_density           0.0    0.0
## Present_cases         0.0    0.0
##
## Error terms:
##  Groups        Name        Std.Dev.
##  FEDUID:Region (Intercept) 0.035
##  Region        (Intercept) 0.087
##  Age_Gender    (Intercept) 0.065
##  Education     (Intercept) 0.164
## Num. levels: FEDUID:Region 329, Region 6, Age_Gender 6, Education 3
##
## ------
## * For help interpreting the printed output see ?print.stanreg
## * For info on the priors used see ?prior_summary.stanreg
```

```
summary(duration_mod_stan)
```

```
##
## Model Info:
##  function:     stan_glmer
##  family:       poisson [log]
##  formula:      Duration ~ (1 | Age_Gender) + (1 | Education) + (1 | Region/FEDUID) +
##     Liberal_voteshare + Inc_per_filer + CCB_dollars_per_recip +
##     Population + Pop_density + Present_cases
##  algorithm:    sampling
##  sample:       12000 (posterior sample size)
##  priors:       see help('prior_summary')
##  observations: 2428
##  groups:       FEDUID:Region (329), Region (6), Age_Gender (6), Education (3)
##
## Estimates:
##                                              mean   sd   10%   50%   90%
```

```
## (Intercept)                                    1.6    0.1  1.5   1.6   1.7
## Liberal_voteshare                              0.0    0.0  0.0   0.0   0.0
## Inc_per_filer                                  0.0    0.0  0.0   0.0   0.0
## CCB_dollars_per_recip                          0.0    0.0  0.0   0.0   0.0
## Population                                     0.0    0.0  0.0   0.0   0.0
## Pop_density                                    0.0    0.0  0.0   0.0   0.0
## Present_cases                                  0.0    0.0 -0.1   0.0   0.0
## b[(Intercept) FEDUID:Region:10001:Atlantic]    0.0    0.0  0.0   0.0   0.0
## b[(Intercept) FEDUID:Region:10002:Atlantic]    0.0    0.0  0.0   0.0   0.0
## b[(Intercept) FEDUID:Region:10003:Atlantic]    0.0    0.0  0.0   0.0   0.0
## b[(Intercept) FEDUID:Region:10005:Atlantic]    0.0    0.0 -0.1   0.0   0.0
## b[(Intercept) FEDUID:Region:10006:Atlantic]    0.0    0.0  0.0   0.0   0.0
## b[(Intercept) FEDUID:Region:10007:Atlantic]    0.0    0.0  0.0   0.0   0.0
## b[(Intercept) FEDUID:Region:11001:Atlantic]    0.0    0.0  0.0   0.0   0.0
## b[(Intercept) FEDUID:Region:11002:Atlantic]    0.0    0.0  0.0   0.0   0.0
## b[(Intercept) FEDUID:Region:11003:Atlantic]    0.0    0.0  0.0   0.0   0.0
## b[(Intercept) FEDUID:Region:11004:Atlantic]    0.0    0.0  0.0   0.0   0.0
## b[(Intercept) FEDUID:Region:12001:Atlantic]    0.0    0.0  0.0   0.0   0.0
## b[(Intercept) FEDUID:Region:12002:Atlantic]    0.0    0.0  0.0   0.0   0.0
## b[(Intercept) FEDUID:Region:12003:Atlantic]    0.0    0.0  0.0   0.0   0.0
## b[(Intercept) FEDUID:Region:12004:Atlantic]    0.0    0.0  0.0   0.0   0.0
## b[(Intercept) FEDUID:Region:12005:Atlantic]    0.0    0.0 -0.1   0.0   0.0
## b[(Intercept) FEDUID:Region:12006:Atlantic]    0.0    0.0  0.0   0.0   0.0
## b[(Intercept) FEDUID:Region:12007:Atlantic]    0.0    0.0  0.0   0.0   0.0
## b[(Intercept) FEDUID:Region:12008:Atlantic]    0.0    0.0  0.0   0.0   0.1
## b[(Intercept) FEDUID:Region:12009:Atlantic]    0.0    0.0  0.0   0.0   0.0
## b[(Intercept) FEDUID:Region:12011:Atlantic]    0.0    0.0  0.0   0.0   0.0
## b[(Intercept) FEDUID:Region:13001:Atlantic]    0.0    0.0  0.0   0.0   0.0
## b[(Intercept) FEDUID:Region:13002:Atlantic]    0.0    0.0  0.0   0.0   0.1
## b[(Intercept) FEDUID:Region:13003:Atlantic]    0.0    0.0 -0.1   0.0   0.0
## b[(Intercept) FEDUID:Region:13004:Atlantic]    0.0    0.0  0.0   0.0   0.0
## b[(Intercept) FEDUID:Region:13005:Atlantic]    0.0    0.0  0.0   0.0   0.0
## b[(Intercept) FEDUID:Region:13006:Atlantic]    0.0    0.0  0.0   0.0   0.0
## b[(Intercept) FEDUID:Region:13007:Atlantic]    0.0    0.0  0.0   0.0   0.0
## b[(Intercept) FEDUID:Region:13008:Atlantic]    0.0    0.0  0.0   0.0   0.0
## b[(Intercept) FEDUID:Region:13009:Atlantic]    0.0    0.0  0.0   0.0   0.1
## b[(Intercept) FEDUID:Region:13010:Atlantic]    0.0    0.0  0.0   0.0   0.0
## b[(Intercept) FEDUID:Region:24001:Quebec]      0.0    0.0  0.0   0.0   0.0
## b[(Intercept) FEDUID:Region:24002:Quebec]      0.0    0.0  0.0   0.0   0.0
## b[(Intercept) FEDUID:Region:24003:Quebec]      0.0    0.0  0.0   0.0   0.0
## b[(Intercept) FEDUID:Region:24004:Quebec]      0.0    0.0  0.0   0.0   0.0
## b[(Intercept) FEDUID:Region:24005:Quebec]      0.0    0.0  0.0   0.0   0.0
## b[(Intercept) FEDUID:Region:24006:Quebec]      0.0    0.0  0.0   0.0   0.0
## b[(Intercept) FEDUID:Region:24007:Quebec]      0.0    0.0 -0.1   0.0   0.0
## b[(Intercept) FEDUID:Region:24008:Quebec]      0.0    0.0  0.0   0.0   0.1
## b[(Intercept) FEDUID:Region:24009:Quebec]      0.0    0.0  0.0   0.0   0.1
## b[(Intercept) FEDUID:Region:24010:Quebec]      0.0    0.0  0.0   0.0   0.0
## b[(Intercept) FEDUID:Region:24011:Quebec]      0.0    0.0  0.0   0.0   0.0
## b[(Intercept) FEDUID:Region:24012:Quebec]      0.0    0.0  0.0   0.0   0.0
## b[(Intercept) FEDUID:Region:24013:Quebec]      0.0    0.0  0.0   0.0   0.0
## b[(Intercept) FEDUID:Region:24014:Quebec]      0.0    0.0  0.0   0.0   0.0
## b[(Intercept) FEDUID:Region:24015:Quebec]      0.0    0.0  0.0   0.0   0.0
## b[(Intercept) FEDUID:Region:24016:Quebec]      0.0    0.0  0.0   0.0   0.0
## b[(Intercept) FEDUID:Region:24017:Quebec]      0.0    0.0  0.0   0.0   0.1
```

```
## b[(Intercept) FEDUID:Region:24018:Quebec]     0.0    0.0  0.0   0.0   0.0
## b[(Intercept) FEDUID:Region:24019:Quebec]     0.0    0.0  0.0   0.0   0.0
## b[(Intercept) FEDUID:Region:24020:Quebec]     0.0    0.0  0.0   0.0   0.0
## b[(Intercept) FEDUID:Region:24021:Quebec]     0.0    0.0  0.0   0.0   0.0
## b[(Intercept) FEDUID:Region:24022:Quebec]     0.0    0.0  0.0   0.0   0.0
## b[(Intercept) FEDUID:Region:24023:Quebec]     0.0    0.0  0.0   0.0   0.0
## b[(Intercept) FEDUID:Region:24024:Quebec]     0.0    0.0  0.0   0.0   0.0
## b[(Intercept) FEDUID:Region:24025:Quebec]     0.0    0.0  0.0   0.0   0.0
## b[(Intercept) FEDUID:Region:24026:Quebec]     0.0    0.0  0.0   0.0   0.0
## b[(Intercept) FEDUID:Region:24027:Quebec]     0.0    0.0 -0.1   0.0   0.0
## b[(Intercept) FEDUID:Region:24028:Quebec]     0.0    0.0  0.0   0.0   0.1
## b[(Intercept) FEDUID:Region:24029:Quebec]     0.0    0.0  0.0   0.0   0.0
## b[(Intercept) FEDUID:Region:24030:Quebec]     0.0    0.0  0.0   0.0   0.0
## b[(Intercept) FEDUID:Region:24031:Quebec]     0.0    0.0  0.0   0.0   0.0
## b[(Intercept) FEDUID:Region:24032:Quebec]     0.0    0.0  0.0   0.0   0.0
## b[(Intercept) FEDUID:Region:24033:Quebec]     0.0    0.0  0.0   0.0   0.0
## b[(Intercept) FEDUID:Region:24034:Quebec]     0.0    0.0  0.0   0.0   0.0
## b[(Intercept) FEDUID:Region:24035:Quebec]     0.0    0.0  0.0   0.0   0.0
## b[(Intercept) FEDUID:Region:24036:Quebec]     0.0    0.0  0.0   0.0   0.0
## b[(Intercept) FEDUID:Region:24037:Quebec]     0.0    0.0 -0.1   0.0   0.0
## b[(Intercept) FEDUID:Region:24038:Quebec]     0.0    0.0  0.0   0.0   0.0
## b[(Intercept) FEDUID:Region:24039:Quebec]     0.0    0.0  0.0   0.0   0.0
## b[(Intercept) FEDUID:Region:24040:Quebec]     0.0    0.0  0.0   0.0   0.0
## b[(Intercept) FEDUID:Region:24041:Quebec]     0.0    0.0  0.0   0.0   0.0
## b[(Intercept) FEDUID:Region:24042:Quebec]     0.0    0.0  0.0   0.0   0.0
## b[(Intercept) FEDUID:Region:24043:Quebec]     0.0    0.0  0.0   0.0   0.0
## b[(Intercept) FEDUID:Region:24044:Quebec]     0.0    0.0  0.0   0.0   0.0
## b[(Intercept) FEDUID:Region:24045:Quebec]     0.0    0.0  0.0   0.0   0.0
## b[(Intercept) FEDUID:Region:24046:Quebec]     0.0    0.0  0.0   0.0   0.0
## b[(Intercept) FEDUID:Region:24047:Quebec]     0.0    0.0 -0.1   0.0   0.0
## b[(Intercept) FEDUID:Region:24048:Quebec]     0.0    0.0  0.0   0.0   0.1
## b[(Intercept) FEDUID:Region:24049:Quebec]     0.0    0.0  0.0   0.0   0.0
## b[(Intercept) FEDUID:Region:24050:Quebec]     0.0    0.0  0.0   0.0   0.0
## b[(Intercept) FEDUID:Region:24051:Quebec]     0.0    0.0  0.0   0.0   0.0
## b[(Intercept) FEDUID:Region:24052:Quebec]     0.0    0.0  0.0   0.0   0.0
## b[(Intercept) FEDUID:Region:24053:Quebec]     0.0    0.0  0.0   0.0   0.0
## b[(Intercept) FEDUID:Region:24054:Quebec]     0.0    0.0  0.0   0.0   0.0
## b[(Intercept) FEDUID:Region:24055:Quebec]     0.0    0.0  0.0   0.0   0.1
## b[(Intercept) FEDUID:Region:24056:Quebec]     0.0    0.0  0.0   0.0   0.0
## b[(Intercept) FEDUID:Region:24057:Quebec]     0.0    0.0  0.0   0.0   0.0
## b[(Intercept) FEDUID:Region:24058:Quebec]     0.0    0.0  0.0   0.0   0.0
## b[(Intercept) FEDUID:Region:24059:Quebec]     0.0    0.0  0.0   0.0   0.0
## b[(Intercept) FEDUID:Region:24060:Quebec]     0.0    0.0  0.0   0.0   0.0
## b[(Intercept) FEDUID:Region:24061:Quebec]     0.0    0.0  0.0   0.0   0.0
## b[(Intercept) FEDUID:Region:24062:Quebec]     0.0    0.0  0.0   0.0   0.0
## b[(Intercept) FEDUID:Region:24063:Quebec]     0.0    0.0  0.0   0.0   0.0
## b[(Intercept) FEDUID:Region:24064:Quebec]     0.0    0.0  0.0   0.0   0.1
## b[(Intercept) FEDUID:Region:24065:Quebec]     0.0    0.0  0.0   0.0   0.0
## b[(Intercept) FEDUID:Region:24066:Quebec]     0.0    0.0  0.0   0.0   0.0
## b[(Intercept) FEDUID:Region:24067:Quebec]     0.0    0.0  0.0   0.0   0.0
## b[(Intercept) FEDUID:Region:24068:Quebec]     0.0    0.0 -0.1   0.0   0.0
## b[(Intercept) FEDUID:Region:24069:Quebec]     0.0    0.0 -0.1   0.0   0.0
## b[(Intercept) FEDUID:Region:24070:Quebec]     0.0    0.0  0.0   0.0   0.1
## b[(Intercept) FEDUID:Region:24071:Quebec]     0.0    0.0  0.0   0.0   0.0
```

```
## b[(Intercept) FEDUID:Region:24072:Quebec]     0.0    0.0  0.0   0.0   0.0
## b[(Intercept) FEDUID:Region:24073:Quebec]     0.0    0.0  0.0   0.0   0.0
## b[(Intercept) FEDUID:Region:24074:Quebec]     0.0    0.0  0.0   0.0   0.0
## b[(Intercept) FEDUID:Region:24075:Quebec]     0.0    0.0  0.0   0.0   0.0
## b[(Intercept) FEDUID:Region:24076:Quebec]     0.0    0.0  0.0   0.0   0.0
## b[(Intercept) FEDUID:Region:24077:Quebec]     0.0    0.0  0.0   0.0   0.0
## b[(Intercept) FEDUID:Region:24078:Quebec]     0.0    0.0  0.0   0.0   0.0
## b[(Intercept) FEDUID:Region:35001:Ontario]    0.0    0.0  0.0   0.0   0.0
## b[(Intercept) FEDUID:Region:35002:Ontario]    0.0    0.0  0.0   0.0   0.0
## b[(Intercept) FEDUID:Region:35003:Ontario]    0.0    0.0  0.0   0.0   0.0
## b[(Intercept) FEDUID:Region:35004:Ontario]    0.0    0.0  0.0   0.0   0.0
## b[(Intercept) FEDUID:Region:35005:Ontario]    0.0    0.0  0.0   0.0   0.0
## b[(Intercept) FEDUID:Region:35006:Ontario]    0.0    0.0  0.0   0.0   0.0
## b[(Intercept) FEDUID:Region:35007:Ontario]    0.0    0.0  0.0   0.0   0.0
## b[(Intercept) FEDUID:Region:35008:Ontario]    0.0    0.0  0.0   0.0   0.0
## b[(Intercept) FEDUID:Region:35009:Ontario]    0.0    0.0  0.0   0.0   0.0
## b[(Intercept) FEDUID:Region:35010:Ontario]    0.0    0.0  0.0   0.0   0.0
## b[(Intercept) FEDUID:Region:35011:Ontario]    0.0    0.0  0.0   0.0   0.0
## b[(Intercept) FEDUID:Region:35012:Ontario]    0.0    0.0  0.0   0.0   0.0
## b[(Intercept) FEDUID:Region:35013:Ontario]    0.0    0.0  0.0   0.0   0.0
## b[(Intercept) FEDUID:Region:35014:Ontario]    0.0    0.0  0.0   0.0   0.0
## b[(Intercept) FEDUID:Region:35015:Ontario]    0.0    0.0  0.0   0.0   0.0
## b[(Intercept) FEDUID:Region:35016:Ontario]    0.0    0.0  0.0   0.0   0.0
## b[(Intercept) FEDUID:Region:35017:Ontario]    0.0    0.0  0.0   0.0   0.0
## b[(Intercept) FEDUID:Region:35018:Ontario]    0.0    0.0 -0.1   0.0   0.0
## b[(Intercept) FEDUID:Region:35019:Ontario]    0.0    0.0  0.0   0.0   0.0
## b[(Intercept) FEDUID:Region:35020:Ontario]    0.0    0.0 -0.1   0.0   0.0
## b[(Intercept) FEDUID:Region:35021:Ontario]    0.0    0.0  0.0   0.0   0.0
## b[(Intercept) FEDUID:Region:35022:Ontario]    0.0    0.0  0.0   0.0   0.0
## b[(Intercept) FEDUID:Region:35023:Ontario]    0.0    0.0 -0.1   0.0   0.0
## b[(Intercept) FEDUID:Region:35024:Ontario]    0.0    0.0  0.0   0.0   0.0
## b[(Intercept) FEDUID:Region:35025:Ontario]    0.0    0.0 -0.1   0.0   0.0
## b[(Intercept) FEDUID:Region:35026:Ontario]    0.0    0.0 -0.1   0.0   0.0
## b[(Intercept) FEDUID:Region:35027:Ontario]    0.0    0.0  0.0   0.0   0.0
## b[(Intercept) FEDUID:Region:35028:Ontario]    0.0    0.0  0.0   0.0   0.0
## b[(Intercept) FEDUID:Region:35029:Ontario]    0.0    0.0  0.0   0.0   0.0
## b[(Intercept) FEDUID:Region:35030:Ontario]    0.0    0.0  0.0   0.0   0.0
## b[(Intercept) FEDUID:Region:35031:Ontario]    0.0    0.0  0.0   0.0   0.0
## b[(Intercept) FEDUID:Region:35032:Ontario]    0.0    0.0  0.0   0.0   0.0
## b[(Intercept) FEDUID:Region:35033:Ontario]    0.0    0.0  0.0   0.0   0.1
## b[(Intercept) FEDUID:Region:35034:Ontario]    0.0    0.0  0.0   0.0   0.0
## b[(Intercept) FEDUID:Region:35035:Ontario]    0.0    0.0  0.0   0.0   0.1
## b[(Intercept) FEDUID:Region:35036:Ontario]    0.0    0.0  0.0   0.0   0.0
## b[(Intercept) FEDUID:Region:35037:Ontario]    0.0    0.0  0.0   0.0   0.0
## b[(Intercept) FEDUID:Region:35038:Ontario]    0.0    0.0 -0.1   0.0   0.0
## b[(Intercept) FEDUID:Region:35039:Ontario]    0.0    0.0 -0.1   0.0   0.0
## b[(Intercept) FEDUID:Region:35040:Ontario]    0.0    0.0  0.0   0.0   0.0
## b[(Intercept) FEDUID:Region:35041:Ontario]    0.0    0.0  0.0   0.0   0.0
## b[(Intercept) FEDUID:Region:35042:Ontario]    0.0    0.0  0.0   0.0   0.0
## b[(Intercept) FEDUID:Region:35043:Ontario]    0.0    0.0  0.0   0.0   0.0
## b[(Intercept) FEDUID:Region:35044:Ontario]    0.0    0.0  0.0   0.0   0.0
## b[(Intercept) FEDUID:Region:35045:Ontario]    0.0    0.0  0.0   0.0   0.0
## b[(Intercept) FEDUID:Region:35046:Ontario]    0.0    0.0  0.0   0.0   0.0
## b[(Intercept) FEDUID:Region:35047:Ontario]    0.0    0.0  0.0   0.0   0.0
```

```
## b[(Intercept) FEDUID:Region:35048:Ontario]    0.0    0.0  0.0   0.0   0.0
## b[(Intercept) FEDUID:Region:35049:Ontario]    0.0    0.0  0.0   0.0   0.0
## b[(Intercept) FEDUID:Region:35050:Ontario]    0.0    0.0  0.0   0.0   0.1
## b[(Intercept) FEDUID:Region:35051:Ontario]    0.0    0.0  0.0   0.0   0.0
## b[(Intercept) FEDUID:Region:35052:Ontario]    0.0    0.0  0.0   0.0   0.0
## b[(Intercept) FEDUID:Region:35053:Ontario]    0.0    0.0  0.0   0.0   0.0
## b[(Intercept) FEDUID:Region:35054:Ontario]    0.0    0.0  0.0   0.0   0.0
## b[(Intercept) FEDUID:Region:35055:Ontario]    0.0    0.0  0.0   0.0   0.1
## b[(Intercept) FEDUID:Region:35056:Ontario]    0.0    0.0  0.0   0.0   0.0
## b[(Intercept) FEDUID:Region:35057:Ontario]    0.0    0.0  0.0   0.0   0.0
## b[(Intercept) FEDUID:Region:35058:Ontario]    0.0    0.0  0.0   0.0   0.0
## b[(Intercept) FEDUID:Region:35059:Ontario]    0.0    0.0  0.0   0.0   0.1
## b[(Intercept) FEDUID:Region:35060:Ontario]    0.0    0.0  0.0   0.0   0.0
## b[(Intercept) FEDUID:Region:35061:Ontario]    0.0    0.0  0.0   0.0   0.1
## b[(Intercept) FEDUID:Region:35062:Ontario]    0.0    0.0 -0.1   0.0   0.0
## b[(Intercept) FEDUID:Region:35063:Ontario]    0.0    0.0  0.0   0.0   0.0
## b[(Intercept) FEDUID:Region:35064:Ontario]    0.0    0.0  0.0   0.0   0.0
## b[(Intercept) FEDUID:Region:35065:Ontario]    0.0    0.0  0.0   0.0   0.1
## b[(Intercept) FEDUID:Region:35066:Ontario]    0.0    0.0  0.0   0.0   0.0
## b[(Intercept) FEDUID:Region:35067:Ontario]    0.0    0.0  0.0   0.0   0.0
## b[(Intercept) FEDUID:Region:35068:Ontario]    0.0    0.0  0.0   0.0   0.1
## b[(Intercept) FEDUID:Region:35069:Ontario]    0.0    0.0  0.0   0.0   0.0
## b[(Intercept) FEDUID:Region:35070:Ontario]    0.0    0.0  0.0   0.0   0.0
## b[(Intercept) FEDUID:Region:35071:Ontario]    0.0    0.0  0.0   0.0   0.1
## b[(Intercept) FEDUID:Region:35072:Ontario]    0.0    0.0  0.0   0.0   0.0
## b[(Intercept) FEDUID:Region:35073:Ontario]    0.0    0.0  0.0   0.0   0.0
## b[(Intercept) FEDUID:Region:35074:Ontario]    0.0    0.0  0.0   0.0   0.0
## b[(Intercept) FEDUID:Region:35075:Ontario]    0.0    0.0  0.0   0.0   0.0
## b[(Intercept) FEDUID:Region:35076:Ontario]    0.0    0.0  0.0   0.0   0.0
## b[(Intercept) FEDUID:Region:35077:Ontario]    0.0    0.0  0.0   0.0   0.1
## b[(Intercept) FEDUID:Region:35078:Ontario]    0.0    0.0  0.0   0.0   0.0
## b[(Intercept) FEDUID:Region:35079:Ontario]    0.0    0.0  0.0   0.0   0.0
## b[(Intercept) FEDUID:Region:35080:Ontario]    0.0    0.0  0.0   0.0   0.1
## b[(Intercept) FEDUID:Region:35081:Ontario]    0.0    0.0  0.0   0.0   0.0
## b[(Intercept) FEDUID:Region:35082:Ontario]    0.0    0.0  0.0   0.0   0.0
## b[(Intercept) FEDUID:Region:35083:Ontario]    0.0    0.0  0.0   0.0   0.0
## b[(Intercept) FEDUID:Region:35084:Ontario]    0.0    0.0  0.0   0.0   0.0
## b[(Intercept) FEDUID:Region:35085:Ontario]    0.0    0.0  0.0   0.0   0.0
## b[(Intercept) FEDUID:Region:35086:Ontario]    0.0    0.0  0.0   0.0   0.0
## b[(Intercept) FEDUID:Region:35087:Ontario]    0.0    0.0  0.0   0.0   0.0
## b[(Intercept) FEDUID:Region:35088:Ontario]    0.0    0.0  0.0   0.0   0.0
## b[(Intercept) FEDUID:Region:35089:Ontario]    0.0    0.0  0.0   0.0   0.0
## b[(Intercept) FEDUID:Region:35090:Ontario]    0.0    0.0  0.0   0.0   0.0
## b[(Intercept) FEDUID:Region:35091:Ontario]    0.0    0.0  0.0   0.0   0.0
## b[(Intercept) FEDUID:Region:35092:Ontario]    0.0    0.0  0.0   0.0   0.0
## b[(Intercept) FEDUID:Region:35093:Ontario]    0.0    0.0  0.0   0.0   0.0
## b[(Intercept) FEDUID:Region:35094:Ontario]    0.0    0.0 -0.1   0.0   0.0
## b[(Intercept) FEDUID:Region:35095:Ontario]    0.0    0.0  0.0   0.0   0.1
## b[(Intercept) FEDUID:Region:35096:Ontario]    0.0    0.0  0.0   0.0   0.0
## b[(Intercept) FEDUID:Region:35097:Ontario]    0.0    0.0  0.0   0.0   0.0
## b[(Intercept) FEDUID:Region:35098:Ontario]    0.0    0.0  0.0   0.0   0.1
## b[(Intercept) FEDUID:Region:35099:Ontario]    0.0    0.0  0.0   0.0   0.0
## b[(Intercept) FEDUID:Region:35100:Ontario]    0.0    0.0  0.0   0.0   0.0
## b[(Intercept) FEDUID:Region:35101:Ontario]    0.0    0.0  0.0   0.0   0.0
```

```
## b[(Intercept) FEDUID:Region:35102:Ontario]    0.0    0.0  0.0   0.0   0.0
## b[(Intercept) FEDUID:Region:35103:Ontario]    0.0    0.0 -0.1   0.0   0.0
## b[(Intercept) FEDUID:Region:35104:Ontario]    0.0    0.0  0.0   0.0   0.0
## b[(Intercept) FEDUID:Region:35105:Ontario]    0.0    0.0  0.0   0.0   0.0
## b[(Intercept) FEDUID:Region:35106:Ontario]    0.0    0.0  0.0   0.0   0.0
## b[(Intercept) FEDUID:Region:35107:Ontario]    0.0    0.0 -0.1   0.0   0.0
## b[(Intercept) FEDUID:Region:35108:Ontario]    0.0    0.0  0.0   0.0   0.0
## b[(Intercept) FEDUID:Region:35109:Ontario]    0.0    0.0  0.0   0.0   0.0
## b[(Intercept) FEDUID:Region:35110:Ontario]    0.0    0.0  0.0   0.0   0.0
## b[(Intercept) FEDUID:Region:35111:Ontario]    0.0    0.0  0.0   0.0   0.0
## b[(Intercept) FEDUID:Region:35112:Ontario]    0.0    0.0  0.0   0.0   0.0
## b[(Intercept) FEDUID:Region:35113:Ontario]    0.0    0.0  0.0   0.0   0.0
## b[(Intercept) FEDUID:Region:35114:Ontario]    0.0    0.0  0.0   0.0   0.0
## b[(Intercept) FEDUID:Region:35115:Ontario]    0.0    0.0  0.0   0.0   0.0
## b[(Intercept) FEDUID:Region:35116:Ontario]    0.0    0.0  0.0   0.0   0.0
## b[(Intercept) FEDUID:Region:35117:Ontario]    0.0    0.0 -0.1   0.0   0.0
## b[(Intercept) FEDUID:Region:35118:Ontario]    0.0    0.0  0.0   0.0   0.0
## b[(Intercept) FEDUID:Region:35119:Ontario]    0.0    0.0  0.0   0.0   0.0
## b[(Intercept) FEDUID:Region:35120:Ontario]    0.0    0.0  0.0   0.0   0.0
## b[(Intercept) FEDUID:Region:35121:Ontario]    0.0    0.0  0.0   0.0   0.0
## b[(Intercept) FEDUID:Region:46001:Prairies]   0.0    0.0  0.0   0.0   0.0
## b[(Intercept) FEDUID:Region:46002:Prairies]   0.0    0.0  0.0   0.0   0.0
## b[(Intercept) FEDUID:Region:46004:Prairies]   0.0    0.0  0.0   0.0   0.0
## b[(Intercept) FEDUID:Region:46005:Prairies]   0.0    0.0  0.0   0.0   0.0
## b[(Intercept) FEDUID:Region:46006:Prairies]   0.0    0.0  0.0   0.0   0.0
## b[(Intercept) FEDUID:Region:46007:Prairies]   0.0    0.0 -0.1   0.0   0.0
## b[(Intercept) FEDUID:Region:46008:Prairies]   0.0    0.0  0.0   0.0   0.0
## b[(Intercept) FEDUID:Region:46009:Prairies]   0.0    0.0  0.0   0.0   0.0
## b[(Intercept) FEDUID:Region:46010:Prairies]   0.0    0.0  0.0   0.0   0.0
## b[(Intercept) FEDUID:Region:46011:Prairies]   0.0    0.0  0.0   0.0   0.1
## b[(Intercept) FEDUID:Region:46012:Prairies]   0.0    0.0  0.0   0.0   0.0
## b[(Intercept) FEDUID:Region:46013:Prairies]   0.0    0.0  0.0   0.0   0.0
## b[(Intercept) FEDUID:Region:46014:Prairies]   0.0    0.0 -0.1   0.0   0.0
## b[(Intercept) FEDUID:Region:47002:Prairies]   0.0    0.0  0.0   0.0   0.0
## b[(Intercept) FEDUID:Region:47003:Prairies]   0.0    0.0  0.0   0.0   0.0
## b[(Intercept) FEDUID:Region:47004:Prairies]   0.0    0.0  0.0   0.0   0.0
## b[(Intercept) FEDUID:Region:47005:Prairies]   0.0    0.0  0.0   0.0   0.0
## b[(Intercept) FEDUID:Region:47006:Prairies]   0.0    0.0  0.0   0.0   0.0
## b[(Intercept) FEDUID:Region:47007:Prairies]   0.0    0.0 -0.1   0.0   0.0
## b[(Intercept) FEDUID:Region:47008:Prairies]   0.0    0.0  0.0   0.0   0.0
## b[(Intercept) FEDUID:Region:47009:Prairies]   0.0    0.0  0.0   0.0   0.1
## b[(Intercept) FEDUID:Region:47010:Prairies]   0.0    0.0  0.0   0.0   0.0
## b[(Intercept) FEDUID:Region:47011:Prairies]   0.0    0.0 -0.1   0.0   0.0
## b[(Intercept) FEDUID:Region:47012:Prairies]   0.0    0.0  0.0   0.0   0.0
## b[(Intercept) FEDUID:Region:47013:Prairies]   0.0    0.0  0.0   0.0   0.1
## b[(Intercept) FEDUID:Region:48001:Alberta]    0.0    0.0  0.0   0.0   0.0
## b[(Intercept) FEDUID:Region:48002:Alberta]    0.0    0.0  0.0   0.0   0.0
## b[(Intercept) FEDUID:Region:48003:Alberta]    0.0    0.0  0.0   0.0   0.0
## b[(Intercept) FEDUID:Region:48004:Alberta]    0.0    0.0  0.0   0.0   0.1
## b[(Intercept) FEDUID:Region:48005:Alberta]    0.0    0.0  0.0   0.0   0.0
## b[(Intercept) FEDUID:Region:48006:Alberta]    0.0    0.0 -0.1   0.0   0.0
## b[(Intercept) FEDUID:Region:48007:Alberta]    0.0    0.0  0.0   0.0   0.0
## b[(Intercept) FEDUID:Region:48008:Alberta]    0.0    0.0  0.0   0.0   0.0
## b[(Intercept) FEDUID:Region:48009:Alberta]    0.0    0.0  0.0   0.0   0.0
```

```
## b[(Intercept) FEDUID:Region:48010:Alberta]    0.0    0.0  0.0   0.0   0.1
## b[(Intercept) FEDUID:Region:48011:Alberta]    0.0    0.0  0.0   0.0   0.0
## b[(Intercept) FEDUID:Region:48012:Alberta]    0.0    0.0  0.0   0.0   0.0
## b[(Intercept) FEDUID:Region:48013:Alberta]    0.0    0.0  0.0   0.0   0.0
## b[(Intercept) FEDUID:Region:48014:Alberta]    0.0    0.0  0.0   0.0   0.0
## b[(Intercept) FEDUID:Region:48015:Alberta]    0.0    0.0  0.0   0.0   0.0
## b[(Intercept) FEDUID:Region:48016:Alberta]    0.0    0.0  0.0   0.0   0.0
## b[(Intercept) FEDUID:Region:48017:Alberta]    0.0    0.0  0.0   0.0   0.0
## b[(Intercept) FEDUID:Region:48018:Alberta]    0.0    0.0  0.0   0.0   0.0
## b[(Intercept) FEDUID:Region:48019:Alberta]    0.0    0.0  0.0   0.0   0.0
## b[(Intercept) FEDUID:Region:48020:Alberta]    0.0    0.0  0.0   0.0   0.0
## b[(Intercept) FEDUID:Region:48021:Alberta]    0.0    0.0  0.0   0.0   0.0
## b[(Intercept) FEDUID:Region:48022:Alberta]    0.0    0.0  0.0   0.0   0.0
## b[(Intercept) FEDUID:Region:48024:Alberta]    0.0    0.0  0.0   0.0   0.0
## b[(Intercept) FEDUID:Region:48025:Alberta]    0.0    0.0  0.0   0.0   0.0
## b[(Intercept) FEDUID:Region:48026:Alberta]    0.0    0.0  0.0   0.0   0.0
## b[(Intercept) FEDUID:Region:48027:Alberta]    0.0    0.0  0.0   0.0   0.1
## b[(Intercept) FEDUID:Region:48028:Alberta]    0.0    0.0  0.0   0.0   0.0
## b[(Intercept) FEDUID:Region:48029:Alberta]    0.0    0.0 -0.1   0.0   0.0
## b[(Intercept) FEDUID:Region:48030:Alberta]    0.0    0.0  0.0   0.0   0.0
## b[(Intercept) FEDUID:Region:48031:Alberta]    0.0    0.0  0.0   0.0   0.0
## b[(Intercept) FEDUID:Region:48032:Alberta]    0.0    0.0  0.0   0.0   0.0
## b[(Intercept) FEDUID:Region:48033:Alberta]    0.0    0.0  0.0   0.0   0.0
## b[(Intercept) FEDUID:Region:48034:Alberta]    0.0    0.0  0.0   0.0   0.0
## b[(Intercept) FEDUID:Region:59001:BC]         0.0    0.0  0.0   0.0   0.1
## b[(Intercept) FEDUID:Region:59002:BC]         0.0    0.0  0.0   0.0   0.0
## b[(Intercept) FEDUID:Region:59003:BC]         0.0    0.0  0.0   0.0   0.0
## b[(Intercept) FEDUID:Region:59004:BC]         0.0    0.0  0.0   0.0   0.0
## b[(Intercept) FEDUID:Region:59005:BC]         0.0    0.0  0.0   0.0   0.0
## b[(Intercept) FEDUID:Region:59006:BC]         0.0    0.0  0.0   0.0   0.0
## b[(Intercept) FEDUID:Region:59007:BC]         0.0    0.0  0.0   0.0   0.0
## b[(Intercept) FEDUID:Region:59008:BC]         0.0    0.0 -0.1   0.0   0.0
## b[(Intercept) FEDUID:Region:59009:BC]         0.0    0.0  0.0   0.0   0.0
## b[(Intercept) FEDUID:Region:59010:BC]         0.0    0.0  0.0   0.0   0.0
## b[(Intercept) FEDUID:Region:59011:BC]         0.0    0.0  0.0   0.0   0.1
## b[(Intercept) FEDUID:Region:59012:BC]         0.0    0.0 -0.1   0.0   0.0
## b[(Intercept) FEDUID:Region:59013:BC]         0.0    0.0  0.0   0.0   0.0
## b[(Intercept) FEDUID:Region:59014:BC]         0.0    0.0  0.0   0.0   0.1
## b[(Intercept) FEDUID:Region:59015:BC]         0.0    0.0  0.0   0.0   0.0
## b[(Intercept) FEDUID:Region:59016:BC]         0.0    0.0  0.0   0.0   0.0
## b[(Intercept) FEDUID:Region:59017:BC]         0.0    0.0  0.0   0.0   0.0
## b[(Intercept) FEDUID:Region:59018:BC]         0.0    0.0  0.0   0.0   0.0
## b[(Intercept) FEDUID:Region:59019:BC]         0.0    0.0  0.0   0.0   0.0
## b[(Intercept) FEDUID:Region:59020:BC]         0.0    0.0 -0.1   0.0   0.0
## b[(Intercept) FEDUID:Region:59021:BC]         0.0    0.0  0.0   0.0   0.0
## b[(Intercept) FEDUID:Region:59022:BC]         0.0    0.0  0.0   0.0   0.1
## b[(Intercept) FEDUID:Region:59023:BC]         0.0    0.0  0.0   0.0   0.1
## b[(Intercept) FEDUID:Region:59024:BC]         0.0    0.0  0.0   0.0   0.0
## b[(Intercept) FEDUID:Region:59025:BC]         0.0    0.0  0.0   0.0   0.0
## b[(Intercept) FEDUID:Region:59026:BC]         0.0    0.0  0.0   0.0   0.0
## b[(Intercept) FEDUID:Region:59027:BC]         0.0    0.0  0.0   0.0   0.0
## b[(Intercept) FEDUID:Region:59028:BC]         0.0    0.0  0.0   0.0   0.0
## b[(Intercept) FEDUID:Region:59029:BC]         0.0    0.0  0.0   0.0   0.0
## b[(Intercept) FEDUID:Region:59030:BC]         0.0    0.0  0.0   0.0   0.0
```

```
## b[(Intercept) FEDUID:Region:59031:BC]         0.0    0.0  0.0   0.0   0.1
## b[(Intercept) FEDUID:Region:59032:BC]         0.0    0.0  0.0   0.0   0.0
## b[(Intercept) FEDUID:Region:59033:BC]         0.0    0.0  0.0   0.0   0.0
## b[(Intercept) FEDUID:Region:59034:BC]         0.0    0.0  0.0   0.0   0.0
## b[(Intercept) FEDUID:Region:59035:BC]         0.0    0.0  0.0   0.0   0.0
## b[(Intercept) FEDUID:Region:59036:BC]         0.0    0.0 -0.1   0.0   0.0
## b[(Intercept) FEDUID:Region:59037:BC]         0.0    0.0  0.0   0.0   0.0
## b[(Intercept) FEDUID:Region:59038:BC]         0.0    0.0  0.0   0.0   0.0
## b[(Intercept) FEDUID:Region:59039:BC]         0.0    0.0  0.0   0.0   0.0
## b[(Intercept) FEDUID:Region:59040:BC]         0.0    0.0  0.0   0.0   0.0
## b[(Intercept) FEDUID:Region:59041:BC]         0.0    0.0  0.0   0.0   0.0
## b[(Intercept) FEDUID:Region:59042:BC]         0.0    0.0  0.0   0.0   0.0
## b[(Intercept) Region:Alberta]                 0.0    0.0 -0.1   0.0   0.0
## b[(Intercept) Region:Atlantic]                0.0    0.1 -0.1   0.0   0.1
## b[(Intercept) Region:BC]                      0.0    0.0  0.0   0.0   0.1
## b[(Intercept) Region:Ontario]                 0.0    0.0  0.0   0.0   0.1
## b[(Intercept) Region:Prairies]                0.0    0.1 -0.1   0.0   0.0
## b[(Intercept) Region:Quebec]                  0.0    0.1 -0.1   0.0   0.1
## b[(Intercept) Age_Gender:18-29_Female]        0.0    0.0 -0.1   0.0   0.0
## b[(Intercept) Age_Gender:18-29_Male]          0.0    0.0  0.0   0.0   0.1
## b[(Intercept) Age_Gender:30-64_Female]        0.0    0.0 -0.1   0.0   0.0
## b[(Intercept) Age_Gender:30-64_Male]          0.0    0.0 -0.1   0.0   0.0
## b[(Intercept) Age_Gender:65+_Female]          0.0    0.0  0.0   0.0   0.1
## b[(Intercept) Age_Gender:65+_Male]            0.0    0.0  0.0   0.0   0.1
## b[(Intercept) Education:Low]                  0.0    0.1  0.0   0.0   0.1
## b[(Intercept) Education:Middle]               0.0    0.1  0.0   0.0   0.1
## b[(Intercept) Education:High]                 0.0    0.1 -0.1   0.0   0.0
## Sigma[FEDUID:Region:(Intercept),(Intercept)]  0.0    0.0  0.0   0.0   0.0
## Sigma[Region:(Intercept),(Intercept)]         0.0    0.0  0.0   0.0   0.0
## Sigma[Age_Gender:(Intercept),(Intercept)]     0.0    0.0  0.0   0.0   0.0
## Sigma[Education:(Intercept),(Intercept)]      0.0    0.2  0.0   0.0   0.0
##
## Fit Diagnostics:
##            mean   sd   10%   50%   90%
## mean_PPD 5.0    0.1  4.9   5.0   5.1
##
## The mean_ppd is the sample average posterior predictive distribution of the outcome variable (for de
##
## MCMC diagnostics
##                                             mcse Rhat n_eff
## (Intercept)                                 0.0  1.0   2877
## Liberal_voteshare                           0.0  1.0  11236
## Inc_per_filer                               0.0  1.0  10967
## CCB_dollars_per_recip                       0.0  1.0  10689
## Population                                  0.0  1.0  10709
## Pop_density                                 0.0  1.0  14457
## Present_cases                               0.0  1.0   3823
## b[(Intercept) FEDUID:Region:10001:Atlantic] 0.0  1.0  12814
## b[(Intercept) FEDUID:Region:10002:Atlantic] 0.0  1.0  13150
## b[(Intercept) FEDUID:Region:10003:Atlantic] 0.0  1.0  12505
## b[(Intercept) FEDUID:Region:10005:Atlantic] 0.0  1.0  11827
## b[(Intercept) FEDUID:Region:10006:Atlantic] 0.0  1.0  14435
## b[(Intercept) FEDUID:Region:10007:Atlantic] 0.0  1.0  14139
## b[(Intercept) FEDUID:Region:11001:Atlantic] 0.0  1.0  13695
```

```
## b[(Intercept) FEDUID:Region:11002:Atlantic]  0.0  1.0  13127
## b[(Intercept) FEDUID:Region:11003:Atlantic]  0.0  1.0  13034
## b[(Intercept) FEDUID:Region:11004:Atlantic]  0.0  1.0  15162
## b[(Intercept) FEDUID:Region:12001:Atlantic]  0.0  1.0  13937
## b[(Intercept) FEDUID:Region:12002:Atlantic]  0.0  1.0  13431
## b[(Intercept) FEDUID:Region:12003:Atlantic]  0.0  1.0  15014
## b[(Intercept) FEDUID:Region:12004:Atlantic]  0.0  1.0  13558
## b[(Intercept) FEDUID:Region:12005:Atlantic]  0.0  1.0   8314
## b[(Intercept) FEDUID:Region:12006:Atlantic]  0.0  1.0  13212
## b[(Intercept) FEDUID:Region:12007:Atlantic]  0.0  1.0  13962
## b[(Intercept) FEDUID:Region:12008:Atlantic]  0.0  1.0   9705
## b[(Intercept) FEDUID:Region:12009:Atlantic]  0.0  1.0  13383
## b[(Intercept) FEDUID:Region:12011:Atlantic]  0.0  1.0  13027
## b[(Intercept) FEDUID:Region:13001:Atlantic]  0.0  1.0  13761
## b[(Intercept) FEDUID:Region:13002:Atlantic]  0.0  1.0  10071
## b[(Intercept) FEDUID:Region:13003:Atlantic]  0.0  1.0  11472
## b[(Intercept) FEDUID:Region:13004:Atlantic]  0.0  1.0  14720
## b[(Intercept) FEDUID:Region:13005:Atlantic]  0.0  1.0  14058
## b[(Intercept) FEDUID:Region:13006:Atlantic]  0.0  1.0  12541
## b[(Intercept) FEDUID:Region:13007:Atlantic]  0.0  1.0  14055
## b[(Intercept) FEDUID:Region:13008:Atlantic]  0.0  1.0  14513
## b[(Intercept) FEDUID:Region:13009:Atlantic]  0.0  1.0   8451
## b[(Intercept) FEDUID:Region:13010:Atlantic]  0.0  1.0  13711
## b[(Intercept) FEDUID:Region:24001:Quebec]    0.0  1.0  11807
## b[(Intercept) FEDUID:Region:24002:Quebec]    0.0  1.0  10761
## b[(Intercept) FEDUID:Region:24003:Quebec]    0.0  1.0  13240
## b[(Intercept) FEDUID:Region:24004:Quebec]    0.0  1.0  14108
## b[(Intercept) FEDUID:Region:24005:Quebec]    0.0  1.0  13758
## b[(Intercept) FEDUID:Region:24006:Quebec]    0.0  1.0  14663
## b[(Intercept) FEDUID:Region:24007:Quebec]    0.0  1.0   6446
## b[(Intercept) FEDUID:Region:24008:Quebec]    0.0  1.0  11408
## b[(Intercept) FEDUID:Region:24009:Quebec]    0.0  1.0  12365
## b[(Intercept) FEDUID:Region:24010:Quebec]    0.0  1.0  14926
## b[(Intercept) FEDUID:Region:24011:Quebec]    0.0  1.0  14407
## b[(Intercept) FEDUID:Region:24012:Quebec]    0.0  1.0  15706
## b[(Intercept) FEDUID:Region:24013:Quebec]    0.0  1.0  12464
## b[(Intercept) FEDUID:Region:24014:Quebec]    0.0  1.0  13513
## b[(Intercept) FEDUID:Region:24015:Quebec]    0.0  1.0  12811
## b[(Intercept) FEDUID:Region:24016:Quebec]    0.0  1.0  13929
## b[(Intercept) FEDUID:Region:24017:Quebec]    0.0  1.0  10049
## b[(Intercept) FEDUID:Region:24018:Quebec]    0.0  1.0  14212
## b[(Intercept) FEDUID:Region:24019:Quebec]    0.0  1.0  11750
## b[(Intercept) FEDUID:Region:24020:Quebec]    0.0  1.0  14233
## b[(Intercept) FEDUID:Region:24021:Quebec]    0.0  1.0  12332
## b[(Intercept) FEDUID:Region:24022:Quebec]    0.0  1.0  13397
## b[(Intercept) FEDUID:Region:24023:Quebec]    0.0  1.0  14604
## b[(Intercept) FEDUID:Region:24024:Quebec]    0.0  1.0  14266
## b[(Intercept) FEDUID:Region:24025:Quebec]    0.0  1.0  12430
## b[(Intercept) FEDUID:Region:24026:Quebec]    0.0  1.0  12352
## b[(Intercept) FEDUID:Region:24027:Quebec]    0.0  1.0  10438
## b[(Intercept) FEDUID:Region:24028:Quebec]    0.0  1.0  12124
## b[(Intercept) FEDUID:Region:24029:Quebec]    0.0  1.0  13675
## b[(Intercept) FEDUID:Region:24030:Quebec]    0.0  1.0  11977
## b[(Intercept) FEDUID:Region:24031:Quebec]    0.0  1.0  14096
```

```
## b[(Intercept) FEDUID:Region:24032:Quebec]    0.0  1.0  14606
## b[(Intercept) FEDUID:Region:24033:Quebec]    0.0  1.0  14443
## b[(Intercept) FEDUID:Region:24034:Quebec]    0.0  1.0  14580
## b[(Intercept) FEDUID:Region:24035:Quebec]    0.0  1.0  12982
## b[(Intercept) FEDUID:Region:24036:Quebec]    0.0  1.0  12287
## b[(Intercept) FEDUID:Region:24037:Quebec]    0.0  1.0  12736
## b[(Intercept) FEDUID:Region:24038:Quebec]    0.0  1.0  11840
## b[(Intercept) FEDUID:Region:24039:Quebec]    0.0  1.0  11027
## b[(Intercept) FEDUID:Region:24040:Quebec]    0.0  1.0  11658
## b[(Intercept) FEDUID:Region:24041:Quebec]    0.0  1.0  14187
## b[(Intercept) FEDUID:Region:24042:Quebec]    0.0  1.0  13853
## b[(Intercept) FEDUID:Region:24043:Quebec]    0.0  1.0  14826
## b[(Intercept) FEDUID:Region:24044:Quebec]    0.0  1.0  15214
## b[(Intercept) FEDUID:Region:24045:Quebec]    0.0  1.0  14302
## b[(Intercept) FEDUID:Region:24046:Quebec]    0.0  1.0  15115
## b[(Intercept) FEDUID:Region:24047:Quebec]    0.0  1.0   9094
## b[(Intercept) FEDUID:Region:24048:Quebec]    0.0  1.0   9639
## b[(Intercept) FEDUID:Region:24049:Quebec]    0.0  1.0  10451
## b[(Intercept) FEDUID:Region:24050:Quebec]    0.0  1.0  15342
## b[(Intercept) FEDUID:Region:24051:Quebec]    0.0  1.0  15962
## b[(Intercept) FEDUID:Region:24052:Quebec]    0.0  1.0  14111
## b[(Intercept) FEDUID:Region:24053:Quebec]    0.0  1.0  14775
## b[(Intercept) FEDUID:Region:24054:Quebec]    0.0  1.0  14891
## b[(Intercept) FEDUID:Region:24055:Quebec]    0.0  1.0  11339
## b[(Intercept) FEDUID:Region:24056:Quebec]    0.0  1.0  13539
## b[(Intercept) FEDUID:Region:24057:Quebec]    0.0  1.0  13938
## b[(Intercept) FEDUID:Region:24058:Quebec]    0.0  1.0  13022
## b[(Intercept) FEDUID:Region:24059:Quebec]    0.0  1.0  11986
## b[(Intercept) FEDUID:Region:24060:Quebec]    0.0  1.0  14377
## b[(Intercept) FEDUID:Region:24061:Quebec]    0.0  1.0  14617
## b[(Intercept) FEDUID:Region:24062:Quebec]    0.0  1.0  14603
## b[(Intercept) FEDUID:Region:24063:Quebec]    0.0  1.0  13666
## b[(Intercept) FEDUID:Region:24064:Quebec]    0.0  1.0   9394
## b[(Intercept) FEDUID:Region:24065:Quebec]    0.0  1.0  13094
## b[(Intercept) FEDUID:Region:24066:Quebec]    0.0  1.0  12981
## b[(Intercept) FEDUID:Region:24067:Quebec]    0.0  1.0  12854
## b[(Intercept) FEDUID:Region:24068:Quebec]    0.0  1.0   9589
## b[(Intercept) FEDUID:Region:24069:Quebec]    0.0  1.0  10779
## b[(Intercept) FEDUID:Region:24070:Quebec]    0.0  1.0   9154
## b[(Intercept) FEDUID:Region:24071:Quebec]    0.0  1.0  12408
## b[(Intercept) FEDUID:Region:24072:Quebec]    0.0  1.0  14816
## b[(Intercept) FEDUID:Region:24073:Quebec]    0.0  1.0  15806
## b[(Intercept) FEDUID:Region:24074:Quebec]    0.0  1.0  13851
## b[(Intercept) FEDUID:Region:24075:Quebec]    0.0  1.0  11682
## b[(Intercept) FEDUID:Region:24076:Quebec]    0.0  1.0  13579
## b[(Intercept) FEDUID:Region:24077:Quebec]    0.0  1.0  14415
## b[(Intercept) FEDUID:Region:24078:Quebec]    0.0  1.0  15740
## b[(Intercept) FEDUID:Region:35001:Ontario]   0.0  1.0  15033
## b[(Intercept) FEDUID:Region:35002:Ontario]   0.0  1.0  13799
## b[(Intercept) FEDUID:Region:35003:Ontario]   0.0  1.0  12505
## b[(Intercept) FEDUID:Region:35004:Ontario]   0.0  1.0  11003
## b[(Intercept) FEDUID:Region:35005:Ontario]   0.0  1.0  14818
## b[(Intercept) FEDUID:Region:35006:Ontario]   0.0  1.0  12872
## b[(Intercept) FEDUID:Region:35007:Ontario]   0.0  1.0  13745
```

```
## b[(Intercept) FEDUID:Region:35008:Ontario]   0.0  1.0  15784
## b[(Intercept) FEDUID:Region:35009:Ontario]   0.0  1.0  13193
## b[(Intercept) FEDUID:Region:35010:Ontario]   0.0  1.0  12143
## b[(Intercept) FEDUID:Region:35011:Ontario]   0.0  1.0  11467
## b[(Intercept) FEDUID:Region:35012:Ontario]   0.0  1.0  13472
## b[(Intercept) FEDUID:Region:35013:Ontario]   0.0  1.0  11528
## b[(Intercept) FEDUID:Region:35014:Ontario]   0.0  1.0  12144
## b[(Intercept) FEDUID:Region:35015:Ontario]   0.0  1.0  12080
## b[(Intercept) FEDUID:Region:35016:Ontario]   0.0  1.0  10841
## b[(Intercept) FEDUID:Region:35017:Ontario]   0.0  1.0  11756
## b[(Intercept) FEDUID:Region:35018:Ontario]   0.0  1.0   7156
## b[(Intercept) FEDUID:Region:35019:Ontario]   0.0  1.0  13697
## b[(Intercept) FEDUID:Region:35020:Ontario]   0.0  1.0   4554
## b[(Intercept) FEDUID:Region:35021:Ontario]   0.0  1.0  13098
## b[(Intercept) FEDUID:Region:35022:Ontario]   0.0  1.0  12856
## b[(Intercept) FEDUID:Region:35023:Ontario]   0.0  1.0   9628
## b[(Intercept) FEDUID:Region:35024:Ontario]   0.0  1.0  14523
## b[(Intercept) FEDUID:Region:35025:Ontario]   0.0  1.0  11853
## b[(Intercept) FEDUID:Region:35026:Ontario]   0.0  1.0   9988
## b[(Intercept) FEDUID:Region:35027:Ontario]   0.0  1.0  13332
## b[(Intercept) FEDUID:Region:35028:Ontario]   0.0  1.0  10820
## b[(Intercept) FEDUID:Region:35029:Ontario]   0.0  1.0  14418
## b[(Intercept) FEDUID:Region:35030:Ontario]   0.0  1.0  11581
## b[(Intercept) FEDUID:Region:35031:Ontario]   0.0  1.0  13646
## b[(Intercept) FEDUID:Region:35032:Ontario]   0.0  1.0  14086
## b[(Intercept) FEDUID:Region:35033:Ontario]   0.0  1.0  10540
## b[(Intercept) FEDUID:Region:35034:Ontario]   0.0  1.0  13154
## b[(Intercept) FEDUID:Region:35035:Ontario]   0.0  1.0   8502
## b[(Intercept) FEDUID:Region:35036:Ontario]   0.0  1.0  14675
## b[(Intercept) FEDUID:Region:35037:Ontario]   0.0  1.0  12005
## b[(Intercept) FEDUID:Region:35038:Ontario]   0.0  1.0   8092
## b[(Intercept) FEDUID:Region:35039:Ontario]   0.0  1.0  12117
## b[(Intercept) FEDUID:Region:35040:Ontario]   0.0  1.0  13880
## b[(Intercept) FEDUID:Region:35041:Ontario]   0.0  1.0  13239
## b[(Intercept) FEDUID:Region:35042:Ontario]   0.0  1.0  11376
## b[(Intercept) FEDUID:Region:35043:Ontario]   0.0  1.0  15338
## b[(Intercept) FEDUID:Region:35044:Ontario]   0.0  1.0  14568
## b[(Intercept) FEDUID:Region:35045:Ontario]   0.0  1.0  14399
## b[(Intercept) FEDUID:Region:35046:Ontario]   0.0  1.0  13610
## b[(Intercept) FEDUID:Region:35047:Ontario]   0.0  1.0  14822
## b[(Intercept) FEDUID:Region:35048:Ontario]   0.0  1.0  14032
## b[(Intercept) FEDUID:Region:35049:Ontario]   0.0  1.0  14117
## b[(Intercept) FEDUID:Region:35050:Ontario]   0.0  1.0  10128
## b[(Intercept) FEDUID:Region:35051:Ontario]   0.0  1.0  13281
## b[(Intercept) FEDUID:Region:35052:Ontario]   0.0  1.0  14569
## b[(Intercept) FEDUID:Region:35053:Ontario]   0.0  1.0  13841
## b[(Intercept) FEDUID:Region:35054:Ontario]   0.0  1.0  13007
## b[(Intercept) FEDUID:Region:35055:Ontario]   0.0  1.0  11939
## b[(Intercept) FEDUID:Region:35056:Ontario]   0.0  1.0  15617
## b[(Intercept) FEDUID:Region:35057:Ontario]   0.0  1.0  13946
## b[(Intercept) FEDUID:Region:35058:Ontario]   0.0  1.0  13591
## b[(Intercept) FEDUID:Region:35059:Ontario]   0.0  1.0   9895
## b[(Intercept) FEDUID:Region:35060:Ontario]   0.0  1.0  14489
## b[(Intercept) FEDUID:Region:35061:Ontario]   0.0  1.0   9265
```

```
## b[(Intercept) FEDUID:Region:35062:Ontario]   0.0  1.0  10866
## b[(Intercept) FEDUID:Region:35063:Ontario]   0.0  1.0  15090
## b[(Intercept) FEDUID:Region:35064:Ontario]   0.0  1.0  15637
## b[(Intercept) FEDUID:Region:35065:Ontario]   0.0  1.0   7080
## b[(Intercept) FEDUID:Region:35066:Ontario]   0.0  1.0  12416
## b[(Intercept) FEDUID:Region:35067:Ontario]   0.0  1.0  13870
## b[(Intercept) FEDUID:Region:35068:Ontario]   0.0  1.0   8034
## b[(Intercept) FEDUID:Region:35069:Ontario]   0.0  1.0  12039
## b[(Intercept) FEDUID:Region:35070:Ontario]   0.0  1.0  14046
## b[(Intercept) FEDUID:Region:35071:Ontario]   0.0  1.0   9054
## b[(Intercept) FEDUID:Region:35072:Ontario]   0.0  1.0  12958
## b[(Intercept) FEDUID:Region:35073:Ontario]   0.0  1.0  13950
## b[(Intercept) FEDUID:Region:35074:Ontario]   0.0  1.0  15004
## b[(Intercept) FEDUID:Region:35075:Ontario]   0.0  1.0  11772
## b[(Intercept) FEDUID:Region:35076:Ontario]   0.0  1.0  11553
## b[(Intercept) FEDUID:Region:35077:Ontario]   0.0  1.0  11100
## b[(Intercept) FEDUID:Region:35078:Ontario]   0.0  1.0  15696
## b[(Intercept) FEDUID:Region:35079:Ontario]   0.0  1.0  12358
## b[(Intercept) FEDUID:Region:35080:Ontario]   0.0  1.0  10802
## b[(Intercept) FEDUID:Region:35081:Ontario]   0.0  1.0  12985
## b[(Intercept) FEDUID:Region:35082:Ontario]   0.0  1.0  11731
## b[(Intercept) FEDUID:Region:35083:Ontario]   0.0  1.0  15159
## b[(Intercept) FEDUID:Region:35084:Ontario]   0.0  1.0  14726
## b[(Intercept) FEDUID:Region:35085:Ontario]   0.0  1.0  15084
## b[(Intercept) FEDUID:Region:35086:Ontario]   0.0  1.0  14238
## b[(Intercept) FEDUID:Region:35087:Ontario]   0.0  1.0  14505
## b[(Intercept) FEDUID:Region:35088:Ontario]   0.0  1.0  11361
## b[(Intercept) FEDUID:Region:35089:Ontario]   0.0  1.0  13562
## b[(Intercept) FEDUID:Region:35090:Ontario]   0.0  1.0  14283
## b[(Intercept) FEDUID:Region:35091:Ontario]   0.0  1.0  13247
## b[(Intercept) FEDUID:Region:35092:Ontario]   0.0  1.0  14972
## b[(Intercept) FEDUID:Region:35093:Ontario]   0.0  1.0  13409
## b[(Intercept) FEDUID:Region:35094:Ontario]   0.0  1.0   9441
## b[(Intercept) FEDUID:Region:35095:Ontario]   0.0  1.0   9164
## b[(Intercept) FEDUID:Region:35096:Ontario]   0.0  1.0  12417
## b[(Intercept) FEDUID:Region:35097:Ontario]   0.0  1.0  13839
## b[(Intercept) FEDUID:Region:35098:Ontario]   0.0  1.0  10086
## b[(Intercept) FEDUID:Region:35099:Ontario]   0.0  1.0  13961
## b[(Intercept) FEDUID:Region:35100:Ontario]   0.0  1.0  13276
## b[(Intercept) FEDUID:Region:35101:Ontario]   0.0  1.0  15007
## b[(Intercept) FEDUID:Region:35102:Ontario]   0.0  1.0  12919
## b[(Intercept) FEDUID:Region:35103:Ontario]   0.0  1.0   8131
## b[(Intercept) FEDUID:Region:35104:Ontario]   0.0  1.0  14829
## b[(Intercept) FEDUID:Region:35105:Ontario]   0.0  1.0  13107
## b[(Intercept) FEDUID:Region:35106:Ontario]   0.0  1.0  13218
## b[(Intercept) FEDUID:Region:35107:Ontario]   0.0  1.0  11648
## b[(Intercept) FEDUID:Region:35108:Ontario]   0.0  1.0  13744
## b[(Intercept) FEDUID:Region:35109:Ontario]   0.0  1.0  13129
## b[(Intercept) FEDUID:Region:35110:Ontario]   0.0  1.0  13407
## b[(Intercept) FEDUID:Region:35111:Ontario]   0.0  1.0  14647
## b[(Intercept) FEDUID:Region:35112:Ontario]   0.0  1.0  13680
## b[(Intercept) FEDUID:Region:35113:Ontario]   0.0  1.0  14579
## b[(Intercept) FEDUID:Region:35114:Ontario]   0.0  1.0  15302
## b[(Intercept) FEDUID:Region:35115:Ontario]   0.0  1.0  15069
```

```
## b[(Intercept) FEDUID:Region:35116:Ontario]   0.0  1.0  14071
## b[(Intercept) FEDUID:Region:35117:Ontario]   0.0  1.0  11110
## b[(Intercept) FEDUID:Region:35118:Ontario]   0.0  1.0  16117
## b[(Intercept) FEDUID:Region:35119:Ontario]   0.0  1.0  14783
## b[(Intercept) FEDUID:Region:35120:Ontario]   0.0  1.0  16893
## b[(Intercept) FEDUID:Region:35121:Ontario]   0.0  1.0  15086
## b[(Intercept) FEDUID:Region:46001:Prairies]  0.0  1.0  13509
## b[(Intercept) FEDUID:Region:46002:Prairies]  0.0  1.0  13504
## b[(Intercept) FEDUID:Region:46004:Prairies]  0.0  1.0  11676
## b[(Intercept) FEDUID:Region:46005:Prairies]  0.0  1.0  12319
## b[(Intercept) FEDUID:Region:46006:Prairies]  0.0  1.0  15016
## b[(Intercept) FEDUID:Region:46007:Prairies]  0.0  1.0  10817
## b[(Intercept) FEDUID:Region:46008:Prairies]  0.0  1.0  13600
## b[(Intercept) FEDUID:Region:46009:Prairies]  0.0  1.0  12885
## b[(Intercept) FEDUID:Region:46010:Prairies]  0.0  1.0  13702
## b[(Intercept) FEDUID:Region:46011:Prairies]  0.0  1.0  10311
## b[(Intercept) FEDUID:Region:46012:Prairies]  0.0  1.0  11546
## b[(Intercept) FEDUID:Region:46013:Prairies]  0.0  1.0  14201
## b[(Intercept) FEDUID:Region:46014:Prairies]  0.0  1.0  10416
## b[(Intercept) FEDUID:Region:47002:Prairies]  0.0  1.0  11447
## b[(Intercept) FEDUID:Region:47003:Prairies]  0.0  1.0  14513
## b[(Intercept) FEDUID:Region:47004:Prairies]  0.0  1.0  13196
## b[(Intercept) FEDUID:Region:47005:Prairies]  0.0  1.0  13800
## b[(Intercept) FEDUID:Region:47006:Prairies]  0.0  1.0  11168
## b[(Intercept) FEDUID:Region:47007:Prairies]  0.0  1.0   9108
## b[(Intercept) FEDUID:Region:47008:Prairies]  0.0  1.0  13174
## b[(Intercept) FEDUID:Region:47009:Prairies]  0.0  1.0   9373
## b[(Intercept) FEDUID:Region:47010:Prairies]  0.0  1.0  14067
## b[(Intercept) FEDUID:Region:47011:Prairies]  0.0  1.0  10991
## b[(Intercept) FEDUID:Region:47012:Prairies]  0.0  1.0  12610
## b[(Intercept) FEDUID:Region:47013:Prairies]  0.0  1.0   9775
## b[(Intercept) FEDUID:Region:48001:Alberta]   0.0  1.0  13783
## b[(Intercept) FEDUID:Region:48002:Alberta]   0.0  1.0  14100
## b[(Intercept) FEDUID:Region:48003:Alberta]   0.0  1.0  14518
## b[(Intercept) FEDUID:Region:48004:Alberta]   0.0  1.0   6162
## b[(Intercept) FEDUID:Region:48005:Alberta]   0.0  1.0  15129
## b[(Intercept) FEDUID:Region:48006:Alberta]   0.0  1.0  11515
## b[(Intercept) FEDUID:Region:48007:Alberta]   0.0  1.0  12521
## b[(Intercept) FEDUID:Region:48008:Alberta]   0.0  1.0  14199
## b[(Intercept) FEDUID:Region:48009:Alberta]   0.0  1.0  15915
## b[(Intercept) FEDUID:Region:48010:Alberta]   0.0  1.0  12862
## b[(Intercept) FEDUID:Region:48011:Alberta]   0.0  1.0  13265
## b[(Intercept) FEDUID:Region:48012:Alberta]   0.0  1.0  13981
## b[(Intercept) FEDUID:Region:48013:Alberta]   0.0  1.0  14329
## b[(Intercept) FEDUID:Region:48014:Alberta]   0.0  1.0  13619
## b[(Intercept) FEDUID:Region:48015:Alberta]   0.0  1.0  14299
## b[(Intercept) FEDUID:Region:48016:Alberta]   0.0  1.0  13393
## b[(Intercept) FEDUID:Region:48017:Alberta]   0.0  1.0  12717
## b[(Intercept) FEDUID:Region:48018:Alberta]   0.0  1.0  11900
## b[(Intercept) FEDUID:Region:48019:Alberta]   0.0  1.0  15050
## b[(Intercept) FEDUID:Region:48020:Alberta]   0.0  1.0  10497
## b[(Intercept) FEDUID:Region:48021:Alberta]   0.0  1.0  12672
## b[(Intercept) FEDUID:Region:48022:Alberta]   0.0  1.0  14004
## b[(Intercept) FEDUID:Region:48024:Alberta]   0.0  1.0  12288
```

```
## b[(Intercept) FEDUID:Region:48025:Alberta]   0.0  1.0  12866
## b[(Intercept) FEDUID:Region:48026:Alberta]   0.0  1.0  14758
## b[(Intercept) FEDUID:Region:48027:Alberta]   0.0  1.0   9843
## b[(Intercept) FEDUID:Region:48028:Alberta]   0.0  1.0  12184
## b[(Intercept) FEDUID:Region:48029:Alberta]   0.0  1.0   8143
## b[(Intercept) FEDUID:Region:48030:Alberta]   0.0  1.0  14662
## b[(Intercept) FEDUID:Region:48031:Alberta]   0.0  1.0  13790
## b[(Intercept) FEDUID:Region:48032:Alberta]   0.0  1.0  13036
## b[(Intercept) FEDUID:Region:48033:Alberta]   0.0  1.0  13431
## b[(Intercept) FEDUID:Region:48034:Alberta]   0.0  1.0  13484
## b[(Intercept) FEDUID:Region:59001:BC]        0.0  1.0   6768
## b[(Intercept) FEDUID:Region:59002:BC]        0.0  1.0  11697
## b[(Intercept) FEDUID:Region:59003:BC]        0.0  1.0  13222
## b[(Intercept) FEDUID:Region:59004:BC]        0.0  1.0  14806
## b[(Intercept) FEDUID:Region:59005:BC]        0.0  1.0  13949
## b[(Intercept) FEDUID:Region:59006:BC]        0.0  1.0  15920
## b[(Intercept) FEDUID:Region:59007:BC]        0.0  1.0  14226
## b[(Intercept) FEDUID:Region:59008:BC]        0.0  1.0   7098
## b[(Intercept) FEDUID:Region:59009:BC]        0.0  1.0  16304
## b[(Intercept) FEDUID:Region:59010:BC]        0.0  1.0  14270
## b[(Intercept) FEDUID:Region:59011:BC]        0.0  1.0  10440
## b[(Intercept) FEDUID:Region:59012:BC]        0.0  1.0   9066
## b[(Intercept) FEDUID:Region:59013:BC]        0.0  1.0  13992
## b[(Intercept) FEDUID:Region:59014:BC]        0.0  1.0  10815
## b[(Intercept) FEDUID:Region:59015:BC]        0.0  1.0  12430
## b[(Intercept) FEDUID:Region:59016:BC]        0.0  1.0  12112
## b[(Intercept) FEDUID:Region:59017:BC]        0.0  1.0  15479
## b[(Intercept) FEDUID:Region:59018:BC]        0.0  1.0  15943
## b[(Intercept) FEDUID:Region:59019:BC]        0.0  1.0  12147
## b[(Intercept) FEDUID:Region:59020:BC]        0.0  1.0   7479
## b[(Intercept) FEDUID:Region:59021:BC]        0.0  1.0  15048
## b[(Intercept) FEDUID:Region:59022:BC]        0.0  1.0  10805
## b[(Intercept) FEDUID:Region:59023:BC]        0.0  1.0  11443
## b[(Intercept) FEDUID:Region:59024:BC]        0.0  1.0  12675
## b[(Intercept) FEDUID:Region:59025:BC]        0.0  1.0  13075
## b[(Intercept) FEDUID:Region:59026:BC]        0.0  1.0  13872
## b[(Intercept) FEDUID:Region:59027:BC]        0.0  1.0   9738
## b[(Intercept) FEDUID:Region:59028:BC]        0.0  1.0  13811
## b[(Intercept) FEDUID:Region:59029:BC]        0.0  1.0  16369
## b[(Intercept) FEDUID:Region:59030:BC]        0.0  1.0  13981
## b[(Intercept) FEDUID:Region:59031:BC]        0.0  1.0   7067
## b[(Intercept) FEDUID:Region:59032:BC]        0.0  1.0  14714
## b[(Intercept) FEDUID:Region:59033:BC]        0.0  1.0  14735
## b[(Intercept) FEDUID:Region:59034:BC]        0.0  1.0  15209
## b[(Intercept) FEDUID:Region:59035:BC]        0.0  1.0  14287
## b[(Intercept) FEDUID:Region:59036:BC]        0.0  1.0   8030
## b[(Intercept) FEDUID:Region:59037:BC]        0.0  1.0  14694
## b[(Intercept) FEDUID:Region:59038:BC]        0.0  1.0  11727
## b[(Intercept) FEDUID:Region:59039:BC]        0.0  1.0  15344
## b[(Intercept) FEDUID:Region:59040:BC]        0.0  1.0  13047
## b[(Intercept) FEDUID:Region:59041:BC]        0.0  1.0  13235
## b[(Intercept) FEDUID:Region:59042:BC]        0.0  1.0  13609
## b[(Intercept) Region:Alberta]                0.0  1.0   7332
## b[(Intercept) Region:Atlantic]               0.0  1.0   6570
```

```r
#multiply each row by its population share per geo unit and sum by geo unit
#country
poststrat_country <- poststrat %>%
  summarize(Duration_pred = sum(duration_pred * Country_percent))

#province
poststrat_prov <- poststrat %>%
  group_by(PRNAME) %>%
  summarize(Duration_pred = sum(duration_pred * Prov_percent)) %>%
  ungroup()

#federal electoral district
poststrat_fed <- poststrat %>%
  group_by(FEDUID) %>%
  summarize(Duration_pred = sum(duration_pred * FED_percent)) %>%
  ungroup()
```

The following block does the poststratification based on the bayesian model. I then examine the full poststratification table before collapsing the table into aggregate units.

```r
#predict outcomes into poststratification table 10,000 times
poststrat <- predicted_draws(duration_mod_stan, newdata = poststrat, allow_new_levels = T,
                             n = 10000, seed = 2684319)
```

Analysis

This block provides the first analysis of the results. It shows the poststratification cells with the highest and lowest (longest and shortest) answers to the question of when almost everyone would be back to work. It then shows the full distribution of mean answers across all 6030 poststratification cells. This reveals that no group expects the almost everyone to be working before July. Finally, I the correspondence between the GLM predictions (which I never use) and the bayesian predictions (which I use throughout).

```r
#groups with longest predicted back to word dates (based on all 6030 cells)
longest_cells <- poststrat %>%
  group_by(Gender, Education, Age, Region, FEDNAME, FEDUID) %>%
  summarize(mean_outcome_bayes = mean(.prediction)) %>%
  ungroup() %>%
  arrange(-mean_outcome_bayes) %>%
  top_n(10, mean_outcome_bayes)

#groups with shortest predicted back to word dates (based on all 6030 cells)
shortest_cells <- poststrat %>%
  group_by(Gender, Education, Age, Region, FEDNAME, FEDUID) %>%
  summarize(mean_outcome_bayes = mean(.prediction)) %>%
  ungroup() %>%
  arrange(mean_outcome_bayes) %>%
  top_n(-10, mean_outcome_bayes)

#histogram of mean predictions for all 6030 cells
#label the highest and lowest ends directly: https://ggplot2-book.org/annotations.html#direct-labelling
poststrat %>%
  group_by(Gender, Education, Age, Region, FEDNAME, FEDUID) %>%
  summarize(mean_outcome_bayes = mean(.prediction)) %>%
  ungroup() %>%
```

```
  ggplot(aes(x = mean_outcome_bayes)) +
  geom_histogram(bins = 50, fill = "light grey") +
  scale_x_continuous(breaks = seq(4, 6.5, 0.5), limits = c(4, 6.5), labels = c("July", "mid-July", "Aug
  theme_minimal() +
  labs(x = "Anticipated date when nearly everyone can go back to work",
       y = "Number of Gender-Age-Education-FED groups") +
  annotate(geom = "curve", x = 4.5, y = 40, xend = 4.23, yend = 2,
           curvature = .2, arrow = arrow(length = unit(2, "mm"))) +
  annotate(geom = "text", x = 4.5, y = 40,
            label = "18-29 year old highly-educated \nwomen living in Beauce, Quebec",
            hjust = "left") +
  annotate(geom = "curve", x = 6.05, y = 100, xend = 6.05, yend = 2,
           curvature = 0, arrow = arrow(length = unit(2, "mm"))) +
  annotate(geom = "text", x = 6.05, y = 120,
            label = "Low-education women older than 65 \nliving in Toronto Centre, Ontario",
            hjust = "center") +
  ggsave("Hist_6030.png", width = 8, height = 6)
```

```
## Warning: Removed 2 rows containing missing values (geom_bar).
```

```
## Warning: Removed 2 rows containing missing values (geom_bar).
```

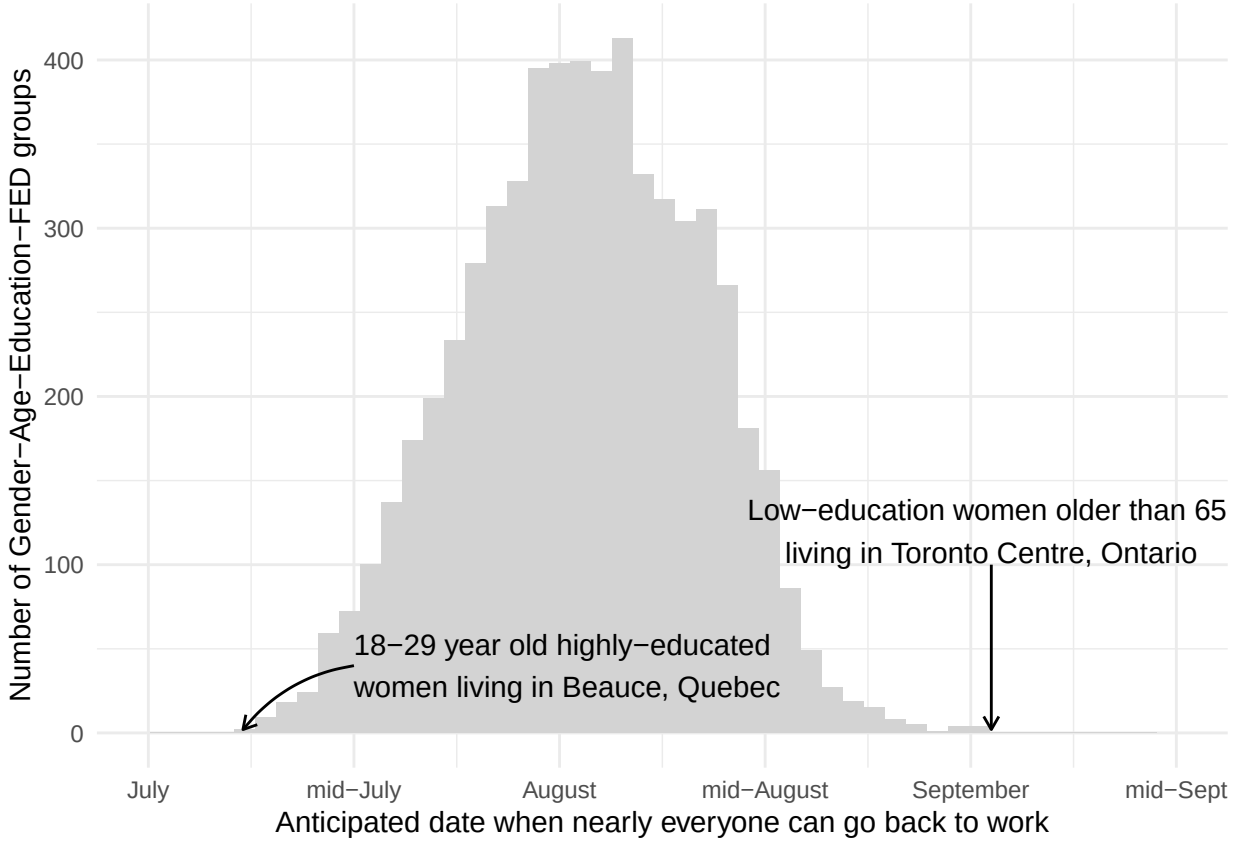


```
#plot means of bayesian draws vs. point predictions from glmer, across all 6030 cells
poststrat %>%
  group_by(.row, duration_pred) %>%
  summarize(mean_outcome_bayes = mean(.prediction)) %>%
  ungroup() %>%
```

```
  ggplot(aes(x = duration_pred, y = mean_outcome_bayes)) +
  geom_point() +
  geom_smooth() +
  labs(x = "GLM prediction",
       y = "Bayesian prediction") +
  geom_abline(intercept = 0, slope = 1, col = "red") +
  theme_minimal() +
  ggsave("GLM_vs_bayes.pdf", width = 6, height = 6)
```

```
## `geom_smooth()` using method = 'gam' and formula 'y ~ s(x, bs = "cs")'
## `geom_smooth()` using method = 'gam' and formula 'y ~ s(x, bs = "cs")'
```

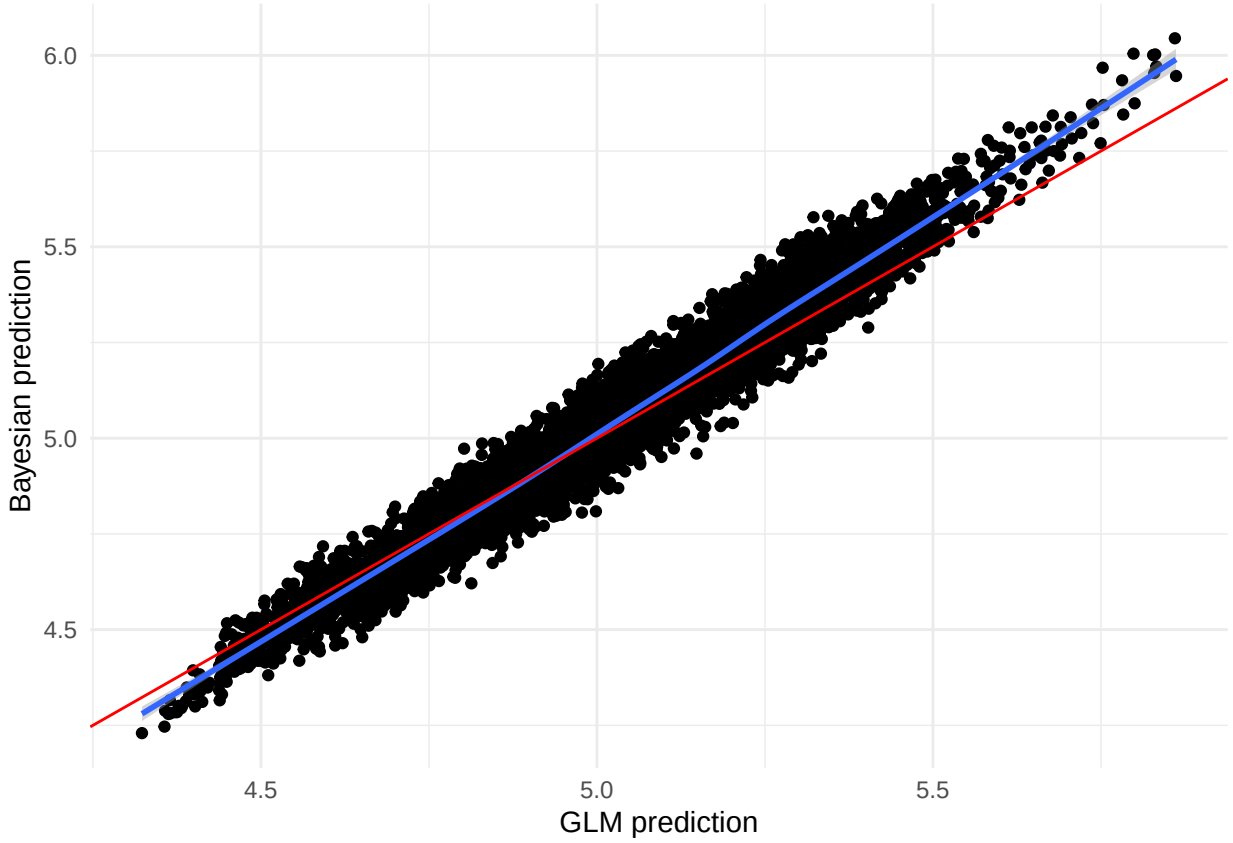

This block makes tables of the key outcomes. It condenses the poststratification table down to the level of the country, then province, then federal electoral district and then shows the mean per group and a 95% predictive interval.

```
#group by row and then calculate weighted .predictions and then ungroup
#and summarize them down to spatial units by draw - so group_by(FEDUID, .draw), which will
#give you 10,000 estimates of the prediction at the lower grouped level

#Country is a special case with no grouping except by .draw
poststrat_country_bayes <- poststrat %>%
  mutate(weighted_prediction = .prediction * Country_percent) %>%
  group_by(.draw) %>%
  summarize(country_pred = sum(weighted_prediction)) %>%
  ungroup() %>%
  summarize(mean = mean(country_pred),
```

```
            lower = quantile(country_pred, 0.025),
            upper = quantile(country_pred, 0.975))

#provincial
poststrat_prov_bayes <- poststrat %>%
  mutate(weighted_prediction = .prediction * Prov_percent) %>%
  group_by(.draw, PRNAME) %>%
  summarize(country_pred = sum(weighted_prediction)) %>%
  ungroup() %>%
  group_by(PRNAME) %>%
  summarize(mean = mean(country_pred),
            lower = quantile(country_pred, 0.025),
            upper = quantile(country_pred, 0.975))

#federal electoral district
poststrat_fed_bayes <- poststrat %>%
  mutate(weighted_prediction = .prediction * FED_percent) %>%
  group_by(.draw, FEDUID) %>%
  summarize(country_pred = sum(weighted_prediction)) %>%
  ungroup() %>%
  group_by(FEDUID) %>%
  summarize(mean = mean(country_pred),
            lower = quantile(country_pred, 0.025),
            upper = quantile(country_pred, 0.975))
```

This makes a plot showing the distribution of post-stratified (weighted) outcomes within each main category of the analysis. For example, when it shows the results by age it takes into consideration that younger people have a different distribution of education than older people. This is the same method that was used to produce estimations into spatial units like provinces above, but here it is used to produce predictions by other categories as well and here the full distribution of 10,000 draws is shown instead of simply the mean and some quantiles.

```
#distributions across provinces
Prov_dist <- poststrat %>%
  mutate(weighted_prediction = .prediction * Prov_percent) %>%
  group_by(.draw, PRNAME) %>%
  summarize(sum_weighted = sum(weighted_prediction),
            variable = "Province") %>%
  ungroup() %>%
  rename(Level_of_var = PRNAME) %>%
  select(variable, Level_of_var, sum_weighted)

#distributions across Canada as a whole
Country_dist <- poststrat %>%
  mutate(weighted_prediction = .prediction * Country_percent) %>%
  group_by(.draw) %>%
  summarize(sum_weighted = sum(weighted_prediction),
            variable = "Country") %>%
  ungroup() %>%
  mutate(Level_of_var = "Canada") %>%
  select(variable, Level_of_var, sum_weighted)

#gender
Gender_dist <- poststrat %>%
```

```
  mutate(weighted_prediction = .prediction * Gender_percent) %>%
  group_by(.draw, Gender) %>%
  summarize(sum_weighted = sum(weighted_prediction),
            variable = "Gender") %>%
  ungroup() %>%
  rename(Level_of_var = Gender) %>%
  select(variable, Level_of_var, sum_weighted)

#age
Age_dist <- poststrat %>%
  mutate(weighted_prediction = .prediction * Age_percent) %>%
  group_by(.draw, Age) %>%
  summarize(sum_weighted = sum(weighted_prediction),
            variable = "Age") %>%
  ungroup() %>%
  rename(Level_of_var = Age) %>%
  select(variable, Level_of_var, sum_weighted)

#education
Education_dist <- poststrat %>%
  mutate(weighted_prediction = .prediction * Education_percent) %>%
  group_by(.draw, Education) %>%
  summarize(sum_weighted = sum(weighted_prediction),
            variable = "Education") %>%
  ungroup() %>%
  rename(Level_of_var = Education) %>%
  select(variable, Level_of_var, sum_weighted)

#graph a grouped dataset of all distributions
grouped <- rbind(Prov_dist, Country_dist, Gender_dist, Age_dist, Education_dist)
#set factor order for plotting
grouped$variable <- factor(grouped$variable,
                           levels = c("Gender", "Age", "Education",
                                      "Province", "Country"))
#set second factor order for plotting
grouped$Level_of_var <- factor(grouped$Level_of_var,
                               levels = c("Male", "Female", "65+", "30-64", "18-29",
                                          "High", "Middle", "Low", "Prince Edward Island",
                                          "British Columbia", "Ontario", "New Brunswick",
                                          "Newfoundland", "Nova Scotia", "Manitoba", "Alberta",
                                          "Saskatchewan", "Quebec", "Canada"))

#calculated 1st percentile of each distribution, then lowest 1st percentile per var
grouped <- grouped %>%
  group_by(Level_of_var) %>%
  mutate(q01 = quantile(sum_weighted, .01)) %>%
  mutate(q99 = quantile(sum_weighted, .99)) %>%
  mutate(mean = mean(sum_weighted)) %>%
  ungroup() %>%
  group_by(variable) %>%
  mutate(minq01 = min(q01)) %>%
  mutate(maxq99 = max(q99)) %>%
  ungroup()
```

```
?stat_halfeyeh
#make main outcome graph
grouped %>%
  ggplot(aes(x = sum_weighted, y = Level_of_var)) +
  stat_halfeyeh(size = 1) +
  #geom_vline(aes(xintercept = minq10, group = Level_of_var), size = 0.5, alpha = 0.4) +
  labs(y = "",
       x = "Anticipated date when nearly everyone can go back to work") +
  facet_grid(rows = vars(variable), scales = "free_y", space = "free_y", switch = "y") +
  theme_minimal() +
  theme(strip.placement = "outside",
        panel.grid.minor = element_blank()) +
  scale_x_continuous(breaks = seq(3.5, 7, 0.5), limits = c(3.5, 7), labels = c("", "July", "", "August"
  ggsave("bayes_groups.pdf", width = 6, height = 8)
```

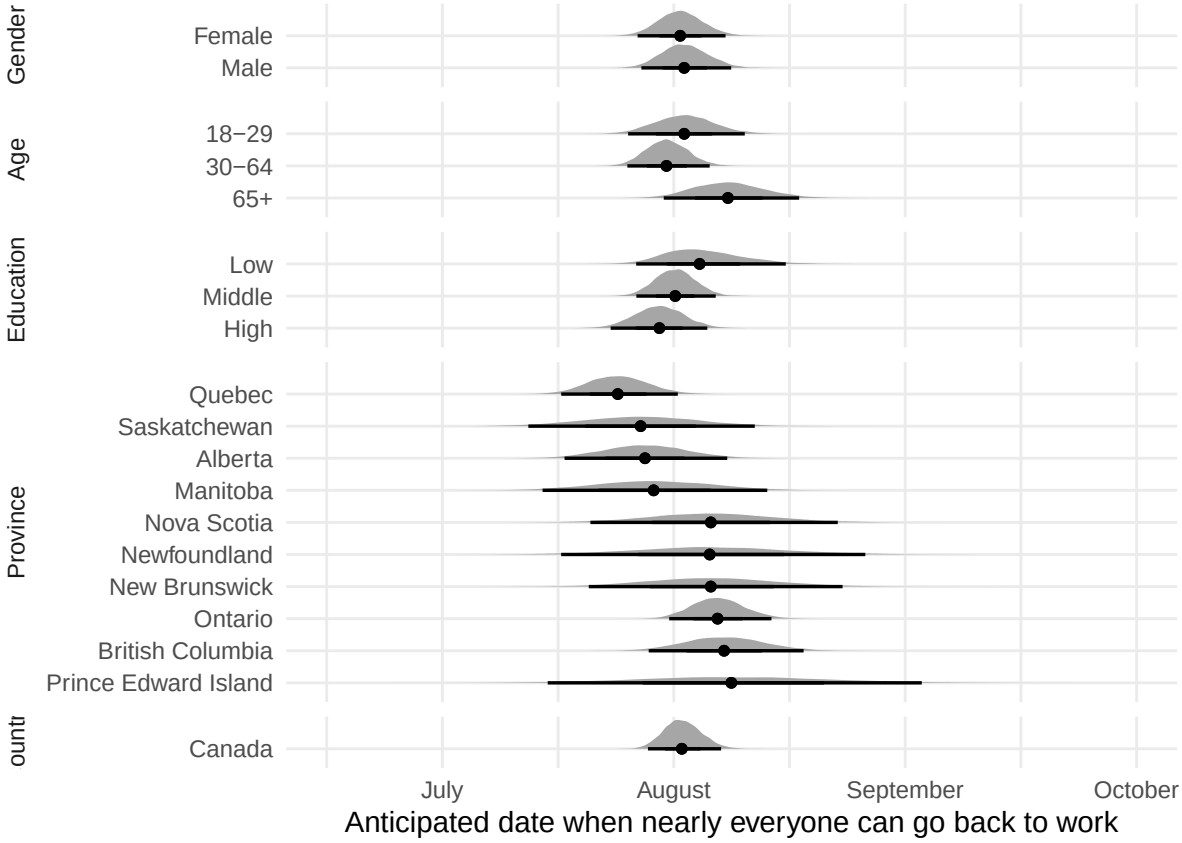

Maps

Finally, I make maps of the results across Canada's federal electoral districts. I lack strong predictors of across-FED variation in the outcome, so this is the weakest part of the analysis. Its main interesting results is the extent to which cities expect longer times before everyone is back to work.

```
#clean map data and merge in predictions
fed <- fed %>%
  filter(!PRNAME %in% c("Yukon", "Nunavut",
                        "Northwest Territories / Territoires du Nord-Ouest")) %>%
  mutate(FEDUID = as.numeric(FEDUID)) %>%
  left_join(poststrat_fed_bayes, by = "FEDUID")
```
